# Deciphering the Structural and Chemical Transformations of Oxide Catalysts during Oxygen Evolution Reaction Using Quick X-ray Absorption Spectroscopy and Machine Learning

**DOI:** 10.1021/jacs.2c11824

**Published:** 2023-02-10

**Authors:** Janis Timoshenko, Felix T. Haase, Sascha Saddeler, Martina Rüscher, Hyo Sang Jeon, Antonia Herzog, Uta Hejral, Arno Bergmann, Stephan Schulz, Beatriz Roldan Cuenya

**Affiliations:** † Department of Interface Science, Fritz-Haber Institute of the Max-Planck Society, 14195 Berlin, Germany; ‡ Institute of Inorganic Chemistry and Center for Nanointegration Duisburg-Essen (CENIDE), University of Duisburg-Essen, 45117 Essen, Germany

## Abstract

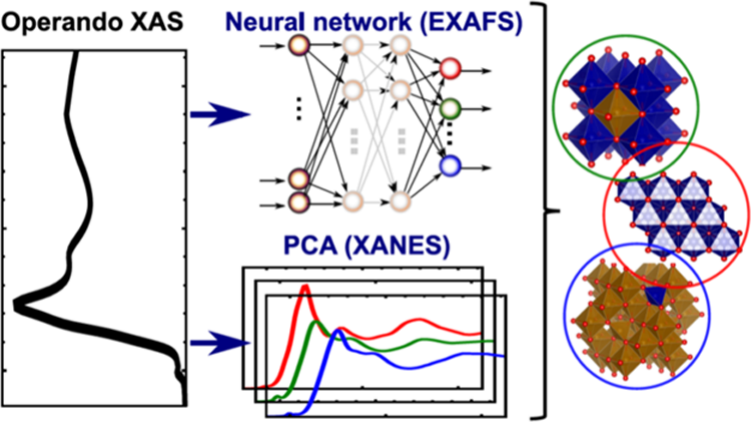

Bimetallic transition-metal oxides, such as spinel-like Co_*x*_Fe_3–*x*_O_4_ materials, are known as attractive catalysts for the oxygen evolution reaction (OER) in alkaline electrolytes. Nonetheless, unveiling the real active species and active states in these catalysts remains a challenge. The coexistence of metal ions in different chemical states and in different chemical environments, including disordered X-ray amorphous phases that all evolve under reaction conditions, hinders the application of common operando techniques. Here, we address this issue by relying on operando quick X-ray absorption fine structure spectroscopy, coupled with unsupervised and supervised machine learning methods. We use principal component analysis to understand the subtle changes in the X-ray absorption near-edge structure spectra and develop an artificial neural network to decipher the extended X-ray absorption fine structure spectra. This allows us to separately track the evolution of tetrahedrally and octahedrally coordinated species and to disentangle the chemical changes and several phase transitions taking place in Co_*x*_Fe_3–*x*_O_4_ catalysts and on their active surface, related to the conversion of disordered oxides into spinel-like structures, transformation of spinels into active oxyhydroxides, and changes in the degree of spinel inversion in the course of the activation treatment and under OER conditions. By correlating the revealed structural changes with the distinct catalytic activity for a series of Co_*x*_Fe_3–*x*_O_4_ samples, we elucidate the active species and OER mechanism.

## 1 Introduction

The oxygen evolution reaction (OER) is the key process for the electrochemical water splitting and production of green hydrogen. It is also central for enabling technologies such as reversible fuel cells and rechargeable metal–air batteries.^1,2^ Wider practical and industrial adoption of these technologies is hindered by the challenges in the design of active and economically viable catalysts. Common OER catalysts are based on iridium and ruthenium metals. These noble materials, which are normally employed in acidic electrolytes, are expensive and may suffer from stability issues (dissolution).^1^ As an attractive alternative, transition-metal oxide catalysts based on earth-abundant metals have been proposed. These catalysts are mainly used in alkaline electrolytes and show by now activities comparable to that of noble catalysts.^1,3,4^ The actual working sites in the oxide catalysts, however, remain often debated. This is especially true for bimetallic oxides, such as Ni_*x*_Fe_3–*x*_O_4_ and Co_*x*_Fe_3–*x*_O_4_, which show the highest OER activity, but where the roles of the different metals (and that of their different possible environments) remain unclear.^2,5,6^

One of the scientific challenges here is the fact that OER is often associated with defective, disordered phases,^1,7^ limiting the applicability of conventional crystallographic approaches for the identification of active states. Furthermore, metal cations with different oxidation states and in different local environments can coexist in these catalysts. This results in a high tunability of these materials but also makes it challenging to understand them using ensemble-averaging methods. Moreover, these metal sites actively respond and partially reversibly evolve under reaction conditions,^8,9^ hence operando time-resolved studies are required to rationalize structure–property relationships in these catalysts. These transformations in OER catalysts, however, are often limited to the near-surface layers of the catalyst only. Thus, the contribution of active species needs to be discerned in the presence of a large fraction of passive spectator species.

Among typical OER catalyst transformations that need to be accounted for are (i) the possible oxidation of metals to higher oxidation states under reaction conditions, (ii) charge redistribution phenomena and the presence of metal complexes having an oxyl radical ligand at the surface (M^*n*+^–O^•^),^10,11^ (iii) transformations of the initial (often amorphous) oxides into thermodynamically more favored spinel structures,^8,9,12,13^ where the distribution of cations between tetrahedrally and octahedrally coordinated sites may vary from one material to another and change under reaction conditions,^12,14^ and (iv) reversible surface-structure transformations of spinels into oxyhydroxide-like structures, where edge-sharing MO_6_ octahedra are believed to be the active sites for the OER.^6,9,15^

These considerations make X-ray absorption fine structure spectroscopy (XAFS) an invaluable tool for tracking the evolution of bimetallic oxide catalysts. Indeed, this element-specific method enables probing the catalyst structure from the perspective of both metals, features high sensitivity to both, the oxidation state of the metals and the local atomistic structure, and can be applied to study materials with any degree of disorder. It is also well suited for time-resolved operando studies within an electrochemical environment.^16^ Indeed, recent developments in quick scanning monochromators and detectors now allow quick XAFS (QXAFS) studies of working electrocatalysts with subsecond time resolution.^17,18^ As a result, the XAFS method has been widely used for probing the active structure of OER catalysts.^4,14,19,20^

While the information encoded in XAFS data is vast, the accurate quantitative deciphering of XAFS spectra remains a non-trivial task due to the aforementioned coexistence of different species, the low spectroscopic contrast between them, and also due to the limitations of conventional data analysis approaches. For example, the determination of the oxidation state for metals is commonly carried out based on the analysis of X-ray absorption near-edge structure (XANES) data. In particular, at X-ray absorption K-edges, higher oxidation states are associated with the shifts of XANES features toward higher energies. However, the definition of the absorption edge position is ambiguous. More importantly, XANES features are affected by changes in the structure, for example, in the distributions of interatomic distances, as well as by the interactions between catalysts and adsorbates.^10^ The effect of structural changes on XANES can be challenging to decouple from that of oxidation state changes. Commonly used XANES analysis approaches for samples with coexisting species, such as linear combination fitting using reference spectra for standard materials, in turn, require to select a-priori a set of relevant references. However, both, the structure and oxidation state of these reference materials can differ significantly from those for metal sites in working OER catalysts. This challenge can in part be addressed by methods like multivariate curve resolution, that aim to deduce the spectra of pure species directly from the experimental data sets.^21−23^ The unambiguity of this approach, however, depends on the particular concentration profiles of the different species, and the results may be biased by the initial guesses, constraints, and assumptions imposed.^23,24^

On the other hand, extended X-ray absorption fine structure (EXAFS) spectra can, in principle, provide quantitative insights into the distribution of atoms around the metal sites. Nonetheless, the analysis of EXAFS features also faces significant challenges, when applied to such complex heterogeneous materials. Indeed, the conventional EXAFS fitting approaches only work reliably for the determination of the nearest neighbor distributions in relatively ordered materials with simple, Gaussian-like distributions of the bond lengths. The common OER oxide catalysts clearly do not belong to this category: coexisting different metal oxidation states, different crystallographic structures, and non-equivalent metal sites present within the same (e.g., spinel-like) structure result in a complicated distribution of interatomic distances, potentially introducing significant systematic errors in the EXAFS fitting [typically underestimated coordination numbers (CNs), interatomic distances, and disorder factors].^16,25^ Considering that metal cations in the relevant oxide materials have similar numbers of nearest neighbors (between 4 and 6), even a small error in the CN determination can lead to wrong conclusions about the speciation of metal sites. Furthermore, the analysis of contributions of distant coordination shells to EXAFS spectra can be more informative for distinguishing between different environments of metal cations. Unfortunately, the accurate accounting for these contributions even in the simplest case of pure spinel materials is a daunting task, requiring to make strong assumptions and imposing constraints on the possible structures, as demonstrated, for example, in the rigorous work by Calvin et al.^26^ In the simple cases where only two different species coexist, differential analysis approaches and linear algebra-based methods can be a viable solution.^27^ However, they do not constitute a general answer for the challenging problem of EXAFS spectra interpretation for the mixtures of different oxide phases evolving under reaction conditions.

To address these problems, and in order to understand the structural and chemical changes in a series of spinel-like Co_*x*_Fe_3–*x*_O_4_ catalysts,^14^ we turn to machine learning (ML)-based approaches. ML already was demonstrated to be a potential breakthrough solution for the analysis of both XANES^22,23,28^ and EXAFS^18,22,29,30^ spectra in heterogeneous materials. In this study, we first employ unsupervised machine learning, namely, principal component analysis (PCA)^22,23,31^ to reveal trends in large sets of QXAFS spectra collected for Co_*x*_Fe_3–*x*_O_4_ catalysts with different Co to Fe ratios and experiencing transformations during the activation treatment and under OER conditions. Our data-driven approach allows us to qualitatively interpret subtle trends in time-resolved XANES without a-priori assumptions about the number of spectroscopically distinct species, and without relying on any empirical assumptions about the relationship between the absorption edge position and chemical state. Second, for the quantitative interpretation of EXAFS data, we develop an artificial neural network (NN) method, which allows reliable analysis of the first and more distant coordination shells. Similar NN-EXAFS approaches have been already previously successfully employed by us for the interpretation of EXAFS spectra both in metallic^22,29,30,32,33^ and oxide^18,22,30,34^ catalysts. However, a key development of the present study is a construction of NN that is able to separately track the evolution of local environments around tetrahedrally and octahedrally coordinated metal sites. This allows us to decouple the effects of different phases, evaluate their transitions, and (through the analysis of interatomic distances), provides independent information on the oxidation of tetrahedrally and octahedrally coordinated Co and Fe cations. Taken together, PCA-XANES and NN-EXAFS results reveal the heterogeneity and complex processes taking place in Co_*x*_Fe_3–*x*_O_4_ catalysts under activation treatment and in the course of OER.

## 2 Experimental and Data Analysis

### 2.1 Catalyst Synthesis, Catalytic Properties, and Prior Characterization

In this work, we focus on three spinel-like nanocatalyst samples with different Co to Fe ratios: Co_2.25_Fe_0.75_O_4_, CoFe_2_O_4_, and Co_0.25_Fe_2.75_O_4_. In addition, an iron-free CoO_*x*_, sample was investigated. The samples were prepared by solvothermal methods. The Co to Fe ratio was systematically varied by changing the molar ratios of the precursors. The synthesis procedure and the results of extensive characterization of these catalysts using electrochemical methods, X-ray diffraction (XRD), electron diffraction, transmission electron microscopy, Mössbauer spectroscopy, magnetometry, X-ray photoelectron spectroscopy, and Fourier-transformed infrared and Raman spectroscopies are elaborated in detail in our previous work.^14^

Briefly, based on these data for the as-prepared samples, it was concluded that the catalyst particle sizes were between 3 and 9 nm, dependently on the Co to Fe ratio (Co-rich particles were smaller than Fe-rich ones). Note here that the small particle sizes in all our samples result in high surface-to-volume ratios and thus facilitate their investigations using sample-averaging spectroscopic methods. It was also shown that the as-prepared bimetallic samples contain a mixture of wüstite-like and spinel-like mixed-metal oxides. The relative fraction of wüstite oxides is the highest in Co-rich samples, while the Co-poor samples are almost phase pure spinels. For the iron-free CoO_*x*_ sample, XRD data suggested the coexistence of rocksalt and wurtzite-type CoO phases in the as-prepared sample. Based on Mössbauer spectroscopy, it was concluded that Fe species do not exhibit strong preference for tetrahedrally or octahedrally coordinated sites in the spinel structure, and in all samples occupy these sites almost randomly. Our OER measurements suggested that the required overpotential decreased with increasing Co content, reaching its minimum for the Co_2.25_Fe_0.75_O_4_ sample. A further increase in the Co content resulted in an increase in the overpotential. See ref (14) for more details.

In that previous work,^14^ we also presented the first results of operando XAFS measurements, which were carried out under steady-state conditions (after ca. 1 h at +1.8 V_RHE_) without tracking the time-dependent evolution of the catalyst [all potential values here are given with respect to the reversible hydrogen electrode (RHE)]. The simplified analysis of those XAFS data included empirical analysis of the absorption edge position and a strongly constrained EXAFS fitting. We emphasize here that such simple data analysis approaches, while providing some useful estimates, cannot fully capture the complexity of these catalysts. Nonetheless, based on these first results, a partially reversible increase in the Co-oxidation state was deduced for all samples under OER conditions but especially for the samples with a higher Co loading. The EXAFS fitting with a strongly constrained fitting model hinted toward a possible increase in the fraction of octahedrally coordinated Co species under OER conditions for the Co-rich samples but not for the Co-poor spinel sample. No significant changes could be detected in the Fe K-edge XAFS data. In the current work, we expand on these prior results and can now conduct a more in-depth analysis, thanks to the acquisition of new time-dependent experimental QXAFS data and the development of more advanced data analysis approaches for their interpretation.

### 2.2 QXAFS Measurements and Analyses

The operando QXAFS measurements were performed at the ROCK beamline at the SOLEIL synchrotron (for Co_2.25_Fe_0.75_O_4_, CoFe_2_O_4_, and Co_0.25_Fe_0.75_O_4_ samples) and at the P64 beamline at PETRA III synchrotron (for the CoO_*x*_ sample). ROCK uses a bending magnet as an X-ray source, while a tapered undulator is used as an X-ray source at P64. Both at the ROCK and the P64 beamline, a channel-cut Si(111) quick scanning monochromator was used for energy selection, a PIPS detector was used for the collection of fluorescence data, while the intensity of incoming X-rays was measured using an ionization chamber filled with nitrogen. The maximal time-resolution of our measurements is determined by the monochromator oscillation frequency, which was set to 2 Hz in the ROCK experiment and 0.5 Hz in the P64 experiment. Furthermore, despite the faster time resolution that can be achieved with these monochromators, for our study several subsequently collected spectra need to be averaged to achieve a signal-to-noise ratio suitable for quantitative analysis. The resulting time resolution of our analysis was thus 50 s.

Operando XAFS measurements were performed in the fluorescence mode at the Fe K-edge (7112 eV) and Co K-edge (7709 eV). Data for both edges were collected for the same sample in a single scan. Measurements in the transmission mode were performed for the reference samples. An in-house built single compartment electrochemical cell^16^ was used for operando XAS measurements. The samples were deposited on the carbon paper (GDE, FuelCellStore) that acted simultaneously as a working electrode and as a window both for the incoming X-rays and for X-ray fluorescence. The sample loading was optimized dependently on the sample composition to ensure the highest possible signal for minority metal, while avoiding self-absorption in the fluorescence XAFS data for the majority metal. A Pt mesh was used as a counter electrode, and a leak-free Ag/AgCl electrode was used as a potential reference. Continuous flow of the electrolyte—0.1 M KOH—was ensured by a peristaltic pump. The following protocol was used for operando measurements: first the samples were measured in the electrolyte under open-circuit potential (OCP) conditions. Next, the samples were activated by cycling the potential 20 times between 1.0 and 1.8 V with 50 mV/s rate. Consequently, samples were exposed to a resting potential of 1.0 V for 15 min, followed by a linear potential ramp from 1.0 to 1.8 V with 1 mV/s rate. The sample was then left under OER conditions (1.8 V) for 15 min, followed by the repeated measurements at the resting potential (1.0 V).

For the ROCK experiment, data calibration and alignment were carried out by aligning the signal from monochromator glitches in operando spectra with those in the reference spectra for Co and Fe foils. For the P64 data, the alignment was carried out using the signal from a Co foil, acquired at the beginning of QXAFS scan, before the sample was moved into the beam. In both cases, the first data alignment, calibration, and averaging were carried out manually using beamline-specific software, followed by automated fine-tuning of the alignment and averaging using a series of home-made *Wolfram Mathematica*(35) scripts. *Wolfram Mathematica* was also used for XANES data extraction and PCA. For EXAFS data extraction, we used *Athena* software,^36^ while the quantitative EXAFS interpretation was carried out based on the neural network method, as described in Section 3.2.

## 3 Results

### 3.1 Principal Component Analysis of XANES Data

**Figure 1:**
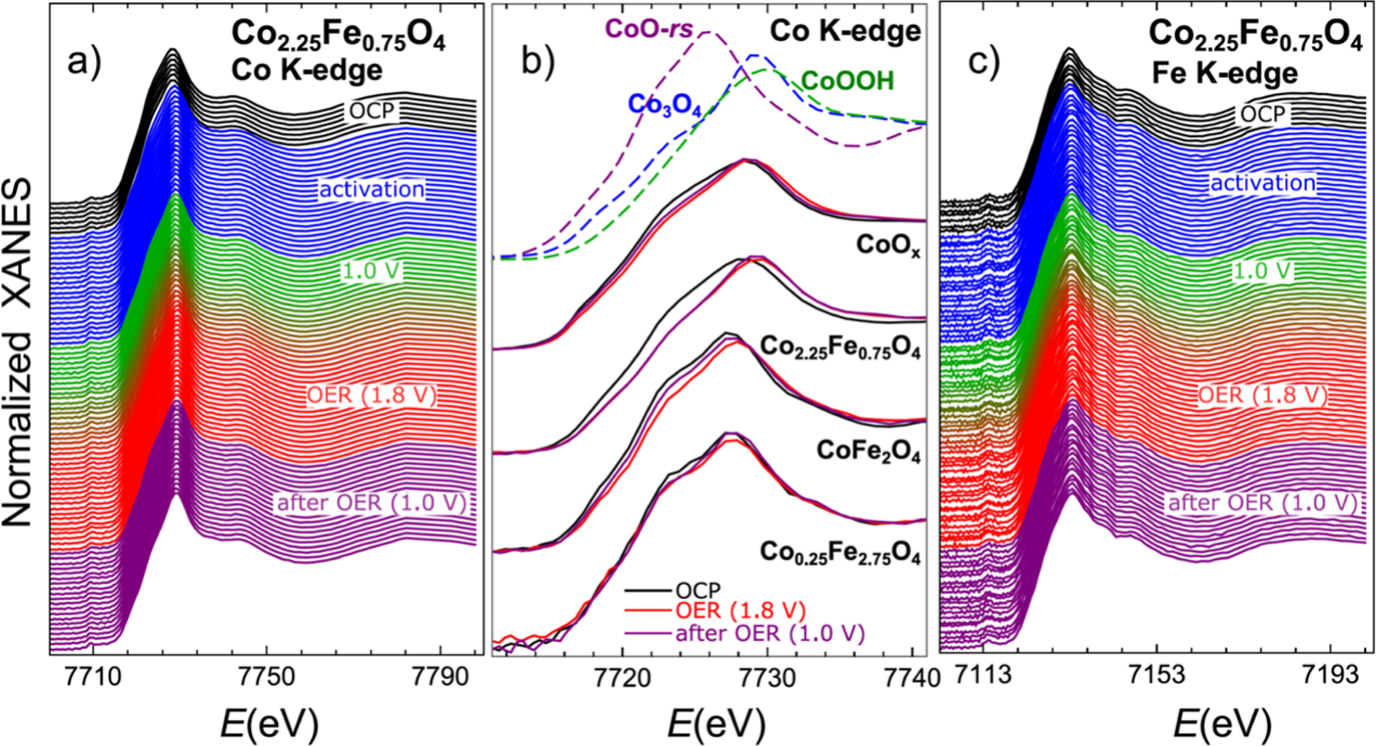
Evolution of the Co K-edge XANES (a,b) and Fe K-edge XANES (c) during activation and under OER conditions for Co_2.25_Fe_0.75_O_4_ (a,c) and CoO_*x*_, CoFe_2_O_4_, and Co_0.25_Fe_2.75_O_4_ (b) nanocatalysts. Spectra are shifted vertically for clarity. (b) Comparison of the representative spectra for the as-prepared samples, samples under OER at 1.8 V and for samples after OER. Each depicted spectrum was obtained after averaging the QXAFS data collected within 50 s.

Time-resolved Co K-edge XANES spectra for CoO_*x*_, Co_2.25_Fe_0.75_O_4_, CoFe_2_O_4_, and Co_0.25_Fe_2.75_O_4_ samples collected before (OCP), during, and after the activation and during and after OER are shown in Figures 1a,b and S1 in the Supporting Information. Overall, all spectra resemble those for Co_3_O_4_ and CoOOH bulk references (Figures 1b and S1f), suggesting a Co-oxidation state between +2 and +3. The changes in the spectra during our electrochemical protocol are rather subtle. Nonetheless, one can notice that during the activation treatment and under OER conditions, the Co K-edge XANES spectra shift toward higher energies, suggesting an increase in the oxidation of the Co species. The oxidation is partially reversible: after OER, when the potential is returned to the resting potential of 1.0 V, the Co K-edge XANES features shift slightly back to the lower energies, although the final spectra do not match those for the as-prepared samples. The reversibility of the chemical state transformations under OER is best seen for the CoFe_2_O_4_ sample (Figures 1b and S1b). The sample with the lowest Co content (Co_0.25_Fe_2.75_O_4_), in turn, exhibits the least pronounced changes under the electrochemical treatment, which, moreover, are irreversible (Figures 1b and S1c).

For a more quantitative discussion, we now turn to the PCA.^23,31,37^ This unsupervised machine learning technique aims to explain the variations in experimental spectroscopic data sets by expressing all the spectra as linear combinations of as few as possible linearly independent components [principal components (PCs)]. The number of PC *d* that is needed to reproduce all the meaningful spectroscopic variations in the experimental data set is related to the number of spectroscopically distinct species present in the data set. A detailed description of our implementation of the PCA method, together with a comparison with the better-known linear combination analysis method is included in the Supporting Information (Supporting Information Note 1) and Figures S1–S6. For the construction of PCs, we used a combined data set containing normalized Co K-edge XANES spectra for CoO_*x*_, Co_2.25_Fe_0.75_O_4_ and CoFe_2_O_4_ (Figures 1a and S1a,b). We did not include here the spectra for Co_0.25_Fe_2.75_O_4_ due to the lower signal-to-noise ratio available for this dilute sample. Nonetheless, once the PCs were obtained, we found that all spectra obtained for Co_0.25_Fe_2.75_O_4_ could also be accurately expressed as linear combination of these PCs (Figure S2), suggesting that this sample does not contain spectroscopically unique species that are not already present in the data sets for CoO_*x*_, Co_2.25_Fe_0.75_O_4_, or CoFe_2_O_4_. More details on the possible relevance of the spectra acquired for the Co_0.25_Fe_2.75_O_4_ sample for the PCs construction are given in Supporting Information Note 1.

**Figure 2:**
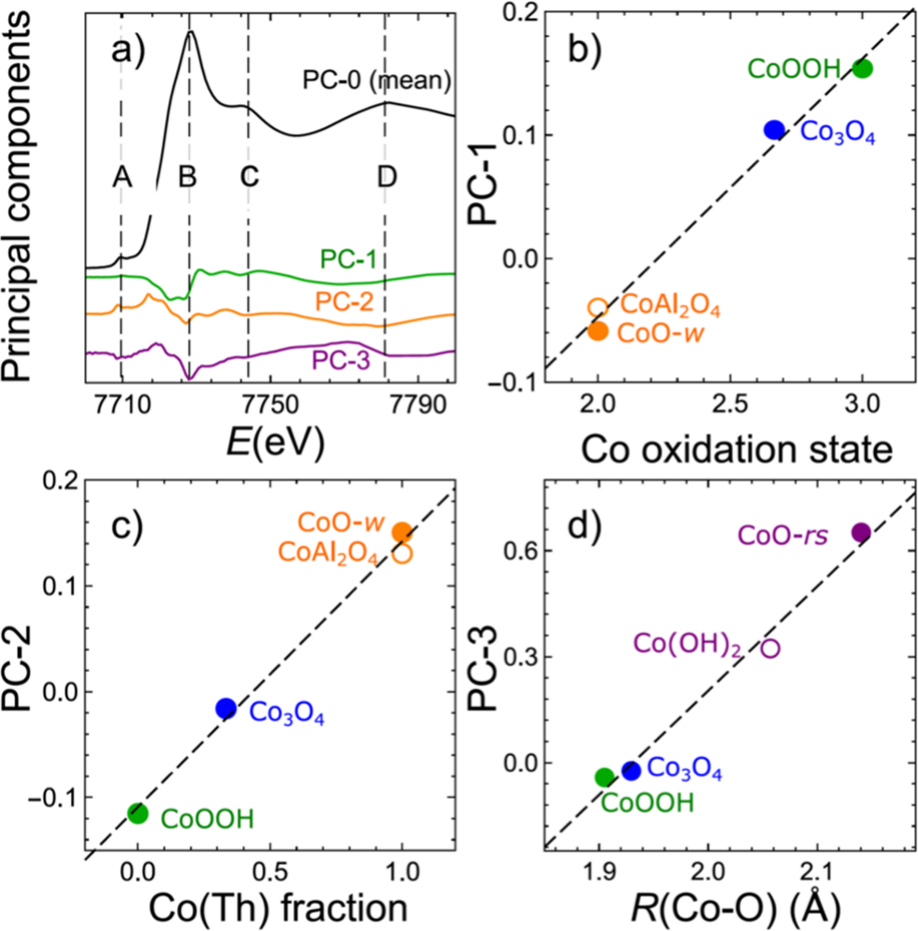
Average spectrum and the first three principal components, as obtained from the combined Co K-edge XANES data set for Co_*x*_Fe_3–*x*_O_4_ catalysts collected under activation conditions as well as during and after OER at +1.8 V_RHE_ (a). Values of the PCs weights to the reference spectra of wurtzite-type and rocksalt-type CoO (CoO-*w* and CoO-*rs*), Co(OH)_2_, CoAl_2_O_4_, Co_3_O_4_, and CoOOH, and their relation to the Co-oxidation state (b), and fraction of Co species in tetrahedral sites (c) and Co–O distance (d).

**Figure 3:**
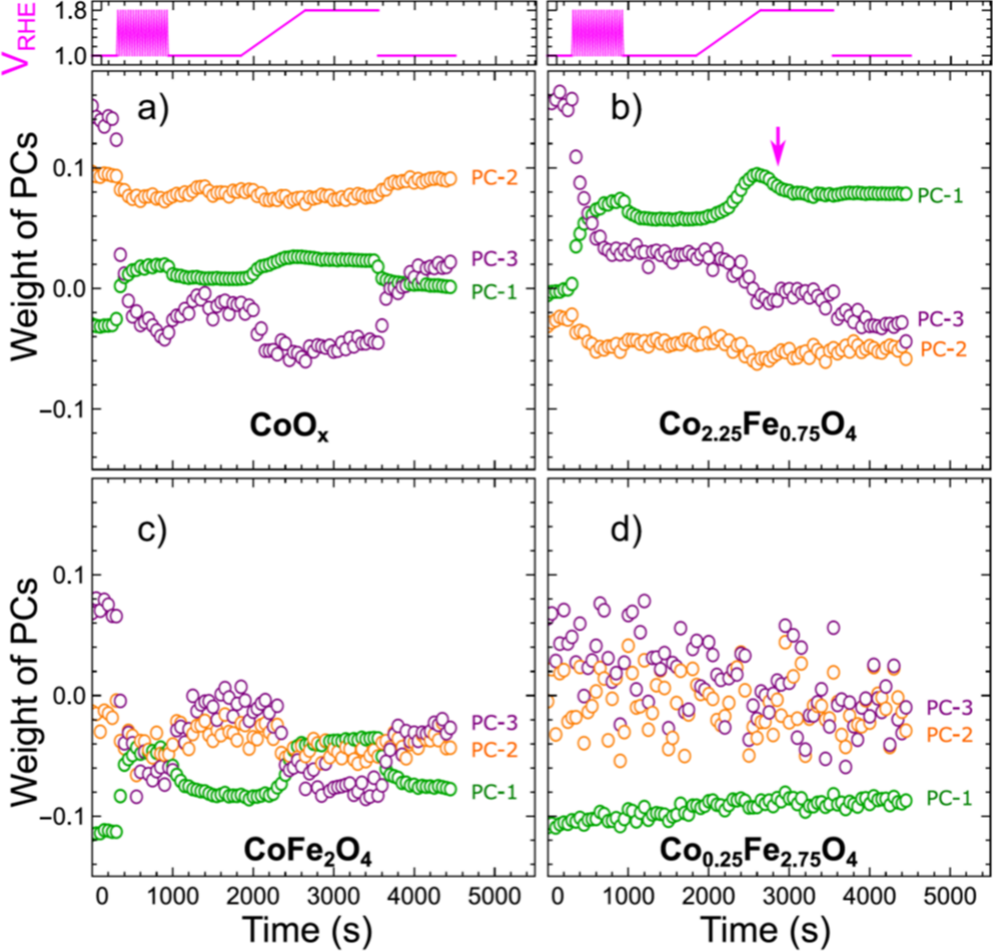
Evolution of the weights of the first, second, and third principal components, as obtained from the combined Co K-edge XANES data set for Co_*x*_Fe_3–*x*_O_4_ catalysts during the activation and under OER conditions at +1.8 V_RHE_ for CoO_*x*_ (a), Co_2.25_Fe_0.75_O_4_ (b), CoFe_2_O_4_ (c), and Co_0.25_Fe_2.75_O_4_ catalysts (d).

The obtained three PCs, indicated as PC-1, PC-2, PC-3, together with the average spectrum (which can be referred to as “PC-0”) are shown in Figure 2. We have found that these PCs are sufficient to describe all the significant variations in our data set. Indeed, we observe that for the PC-1, PC-2, and PC-3, the projections of the experimental spectra on the corresponding PCs (coefficients *a*_*ij*_ in Supporting Information eq S1) change systematically with the changes in the electrochemical conditions and also between different samples (Figure 3). This is not the case for the fourth PC (“PC-4”, see Figure S3) and further PCs. From another perspective, to find the minimal number of PCs required, for each spectrum one can also calculate the residual of the PC decomposition, defined as Euclidean distance between the spectrum and its reconstruction using a given number of PCs *d*. One can see (Figure S4) that the decomposition residual decreases, when *d* is increased from 0 to 3. A further increase of the *d* value, however, does not improve the residual for any of the experimental spectra, and the decomposition error is now dominated by random noise, thus confirming that three PCs are sufficient to describe the physically meaningful information in the entire data set. Furthermore, the so called scree plot, showing the importance of the PCs in terms of the corresponding singular values, also exhibits a clear inflection point at the singular value for the third component, which is a commonly used indicator of the number of necessary PCs for spectra reconstruction (Figure S3b).^23^ Here, we can also note that for *d* values between 1 and 3, the increase of *d* results in different degree of residual improvement for different spectra. For example, the inclusion of the PC-3 mostly improves the residual for the spectra for the as-prepared Co-rich samples at OCP before the activation treatment (Figure S4c,d). This observation shows that certain species do not contribute to the entire data set but are transiently present in our samples.

The fact that four vectors (PC-0, PC-1, PC-2, and PC-3) linearly span the entire data set, shows that only four spectroscopically distinct species are present. For the considered complex system this is rather low. Indeed, as shown above, based on ex situ characterization and on literature data for analogous systems, one could expect that our data set contains contributions of, for instance, Co^2+^ species in rocksalt-like octahedral environment, Co^2+^ species in wurtzite-like tetrahedral environment, Co^2+^ and Co^3+^ species in spinel-like octahedral and tetrahedral environments, and Co^3+^ (and, perhaps, even more oxidized) species in an octahedral CoOOH-like environment. At this point, one can thus conclude that either some of these candidate species are not present in our samples, or, more likely, some of these species produce very similar spectra and cannot be distinguished based only on XANES data.

PCs themselves do not correspond to spectra of any pure species. However, in this particular case, it is possible to deduce what kind of structural or chemical transformations signifies the increase or decrease of a certain PC weight. One can conveniently consider the PC-1, PC-2, and PC-3 as corrections to the averaged spectrum (PC-0). The latter is dominated by four features (Figure 2a): the pre-edge feature A at ca. 7710 eV, the main absorption jump and a “white line” feature B at ca. 7728 eV, post-edge feature C at ca. 7744 eV, and a broad feature D at ca. 7781 eV. One can notice from Figure 2a that the inclusion of PC-1 will have the strongest impact in the spectral region below feature B. Namely, a positive weight of PC-1 will reduce the absorption below feature B and, thus, will result in the shift of the main absorption jump to higher energies. Thus, the positive weight of PC-1 in a Co K-edge XANES spectrum indicates that this spectrum corresponds to a sample with a higher oxidation state of Co than the average oxidation state in the entire data set. On the other hand, the PC-2 and PC-3 have an opposite effect on the absorption edge position to that of PC-1. The positive weights for PC-2 and PC-3 would move the spectra to lower energies. These PCs thus account for the presence of mostly divalent Co species. An important difference between the PC-2 and PC-3 is the pre-edge feature. While the positive weight of PC-2 increases the pre-edge intensity, the positive weight of PC-3 decreases it. Considering that the pre-edge intensity is strongly linked to the contribution of tetrahedrally coordinated species,^38^ the weight of PC-2 to a large extent reflects the concentration of tetrahedral Co^2+^ species. On the other hand, the contribution of PC-3 is the most significant for the as-prepared Co-rich samples at OCP, while it is pronounced neither for the samples after activation nor for the Co-poor samples.

Based on the ex situ XRD data, we know that the as-prepared samples feature the contribution of rocksalt-like CoO species, whose fraction decreases with decreasing Co content. This suggests that PC-3 mostly tracks the Co^2+^ species in an octahedral rocksalt-like environment. We note that, in addition to reducing the pre-edge feature, PC-3 also shifts feature D to lower energies, which could be interpreted as an increase of the Co–O bond lengths.^39^

Furthermore, we have noticed that several standard Co K-edge XANES spectra for Co bulk reference compounds can reasonably be well expressed as a linear combination PC-0, PC-1, PC-2, and PC-3 (Figure S5). This allows us to further validate our interpretation of different PCs, by correlating the projections of the standard Co K-edge XANES spectra on PC-0, PC-1, PC-2, and PC-3 with the known descriptors of the structure and Co chemical state in these reference materials. We observe that in agreement with our considerations above, for spinel-like and CoOOH-like reference spectra, there is a linear correlation between the weight of PC-1 and the Co-oxidation state (Figure 2b). The weight of PC-2, in turn, is nearly linearly correlated with the fraction of Co species in tetrahedral environment (Figure 2c). Finally, the weight of PC-3 can be used to track Co–O distance in a broad range of Co materials (Figure 2d).

PCA reveals important differences in the evolution of the Co-oxidation state and heterogeneous structures of Co_*x*_Fe_3–*x*_O_4_ samples with different Co contents. We first note that the average Co-oxidation state is higher for samples with higher Co concentration (Co_2.25_Fe_0.75_O_4_ and CoO_*x*_), but, nonetheless, in all samples, it is lower than the average oxidation state of Co in Co_3_O_4_ (as evident from comparing the corresponding PC-1 weights, see Figures 2b and 3). In Co_2.25_Fe_0.75_O_4_, the excess Co^2+^ species emanates from the presence of a rocksalt-type cobalt oxide structures, while in the CoO_*x*_ sample, we observe both, rocksalt-type cobalt oxides and oxides with tetrahedral Co coordination (like, e.g., in wurtzite-type CoO). The presence of the latter is indicated by the positive PC-2 contribution, which is unique for the CoO_*x*_ sample. The contribution of this species in the CoO_*x*_ sample is, to a large extent, unchanged during the electrochemical treatment, suggesting that it is weakly involved in the OER process. On the other hand, rocksalt-like species are irreversibly oxidized during the activation treatment, as suggested by the decrease of the PC-3 component for all Co-rich samples during the activation treatment. In addition to these irreversible structural changes, the activation and exposure to the OER conditions in our samples are paralleled by a reversible increase in the Co-oxidation state (changes in PC-1 weight). An exception is the Co-poor Co_0.25_Fe_2.75_O_4_ sample, for which no reversible transformations are observed. Instead, for this sample, we observe a slow continuous increase in the Co-oxidation state, which can be a result of sample interactions with the X-ray beam, or just originate from the exposure to the electrolyte. Indeed, for all samples (with a noteworthy exception being CoFe_2_O_4_), we observe that the intensity of the Co K-edge fluorescence signal diminished with time, which indicates leaching of Co species (Figure S7). This effect, however, seems to be relatively minor in comparison with the pronounced changes in the Co-oxidation state observed for other our investigated catalysts under the activation treatment and during the OER.

The partial dissolution of the catalyst could also explain another intriguing feature, indicated in Figure 3b with a magenta arrow. Namely, we observe that for Co_2.25_Fe_0.75_O_4_ during ramping the potential from the resting potential of 1.0 V to the OER potential of 1.8 V, the Co-oxidation state increases (the weight of PC-1 increases). However, when the stable OER conditions reached, we observe a decrease in the PC-1 weight, despite the fact that the sample is still exposed to 1.8 V potential. Considering that this process coincides with a more rapid dissolution of Co in the Co_2.25_Fe_0.75_O_4_ sample (as indicated by the decrease in the Co fluorescence signal, Figure S7), this could be attributed to the fact that the Co species formed in a high oxidation state are not stable under OER conditions. We note that such a peculiar behavior is less pronounced for the contributions of PC-2 and PC-3 to the spectra of this sample.

Variations in the operando Fe K-edge XANES data are much smaller than those for Co K-edge XANES (Figures 1c and S8). In fact, we observe that all Fe K-edge XANES spectra for all samples strongly resemble the reference spectrum for spinel-type γ-Fe_2_O_3_ oxide (Figure S9). The latter reference spectrum itself resembles the spectrum for Fe_3_O_4_, but is shifted to higher energy, reflecting the higher oxidation state of Fe. We thus can conclude that in all of our samples, Fe is present in a predominantly 3+ state and in a spinel-like environment.

Nonetheless, we observe that the Fe K-edge XANES spectra for samples with different Fe to Co ratios are slightly different (Figure S9). PCA, analogous to that carried out for the Co K-edge data, suggests that the differences between the Fe K-edge XANES are caused by the differences in the concentrations of three co-existing spectroscopically distinct species (Figure S10). We first note that the average Fe K-edge XANES spectrum (PC-Fe-0) shows typical features of spinel-like materials, including the pre-edge A at ca. 7114 eV, the shoulder feature B at ca. 7123 eV, and three characteristic peaks C, D, and E in the white-line region at ca. 7133, 7139, and 7148 eV, respectively. As a correction to this shape, the PCs (PC-Fe-1 and PC-Fe-2) need to be considered (Figure S10a). We note that the positive weight of PC-Fe-1 increases the intensity of the pre-edge features A and B, while suppressing the intensity of the main “white line” feature C. From comparison with the Fe K-edge refence spectra (Figure S9c) and from theoretical simulations of XANES spectra,^40^ we can assign these characteristics to the presence of tetrahedrally coordinated Fe sites. Indeed, ref (40) nicely shows that the enhanced intensity of features A and B and the lower intensity of feature C are the main differences between the XANES spectra for tetrahedrally and octahedrally coordinated Fe sites in spinel-like structures. The differences in the PC-Fe-1 weights for different Co_*x*_Fe_3–*x*_O_4_ spinels thus can be linked to different occupations of tetrahedral and octahedral spinel sites by Fe species. At the same time, as one can see from Figure S10a, the second PC, PC-Fe-2, is responsible for a shift of all Fe K-edge XANES features: the positive contribution of PC-Fe-2 results in the shift of features to higher energies. Thus, PC-Fe-2 can be associated with the changes in the Fe-oxidation state.

PC-Fe-1 accounts for the most differences between the Fe K-edge XANES spectra for different Co_*x*_Fe_3–*x*_O_4_ samples. Based on the arguments above, we conclude that the concentration of Fe in tetrahedral sites increases with the Fe to Co ratio and is the highest for the Co_0.25_Fe_2.75_O_4_ sample. It is important to note that for none of the samples, the PC-Fe-1 weight to Fe K-edge XANES shows any significant variation during the activation or during the OER. This suggests that no major redistribution of Fe species over tetrahedral and octahedral sites in spinel-like structure takes place under OER conditions.

As mentioned above, already in the as-prepared samples, the oxidation state of Fe is close to 3+. For the Co-poor Co_0.25_Fe_2.75_O_4_ sample, no significant further changes in the Fe-oxidation state were observed, as evidenced by the lack of changes in the PC-Fe-2 weight (Figure S10b). Nonetheless, importantly, for the Co_2.25_Fe_0.75_O_4_ and CoFe_2_O_4_ samples, unlike the PC-Fe-1 weight, the PC-Fe-2 weight does change under reaction conditions, revealing that the Fe species are oxidized during the activation and under OER conditions (Figure S10c,d), similar to the Co species. Thus, the importance of Fe to the OER mechanism cannot be ruled out. We highlight here that the increase in Fe-oxidation under OER conditions is much lower than that observed for Co. Even though these changes are hardly visible in the raw XANES data, the PCA approach enables their detection.

Finally, we note that the changes in the Fe K-edge fluorescence intensity (Figure S7b) are similar to those observed at Co K-edge, suggesting that the dissolution of Fe and Co are proportional, and that the ratio between Co and Fe species likely remains close to the nominal one (Figure S7c). A slightly higher Fe dissolution is observed for the CoFe_2_O_4_ sample, resulting in a slightly more Co-rich composition after electrochemical treatment, but this effect is rather small.

### 3.2 Neural Network-Based Method for Deciphering EXAFS Data

While XANES is very useful for tracking the changes in the chemical state, additional detailed information about the structural transformations can be extracted from EXAFS data. For the EXAFS data analysis, in this study, we rely on a neural-network based supervised machine learning approach. We construct a mathematical model and train it to map the relationship between the features in our EXAFS spectra and the partial bond length distributions [partial radial distribution functions (RDFs)] in the heterogeneous materials.

The one-to-one relationship between RDFs and EXAFS (see Note 2 and eq S2 in Supporting Information) means that the problem is invertible, and that the sets of partial RDFs can be, in principle, unambiguously extracted from EXAFS spectra for mixtures. A neural network approach is an efficient solution to this problem. Previously, we have used the NN method to extract from EXAFS data partial RDFs of different metals in bimetallic catalysts,^32^ partial metal–metal and metal oxide contributions in mixed metal/oxide systems,^30,34^ and, recently, partial RDFs corresponding to fcc and non-fcc-type metals.^18^ In this study, we exploit this approach to extract for the first time separate partial site-specific RDFs for metal–oxygen and metal–metal bonds for tetrahedrally and octahedrally coordinated absorbing metal atoms. To this goal, we construct a large set of EXAFS spectra for mixtures, for which the corresponding true RDFs are known. Following the approach introduced in our previous studies,^18,22,30,34^ we first generate pairs of theoretical EXAFS spectra and corresponding RDFs for all unique metal sites in a set of relevant Co and Fe oxides, hydroxides, and oxyhydroxides. In our case, based on the results from ex situ characterization and XANES analysis, we have considered octahedrally coordinated Co species in rocksalt-type Co(II) oxide (CoO-*rs*), Co(OH)_2_ and CoOOH, tetrahedrally coordinated Co species in wurtzite-type CoO (CoO-*w*), as well as tetrahedrally and octahedrally coordinated Co sites in Co_3_O_4_, octahedrally coordinated Fe species in FeO, α-Fe_2_O_3_, FeOOH, and tetrahedrally and octahedrally coordinated Fe sites in spinel-type Fe_3_O_4_ and γ-Fe_2_O_3_. For each of these well-defined reference compounds, we sampled atomic configurations using molecular dynamics or Monte Carlo methods with empirical force field models, keeping also track of whether the central metal atom has an octahedral or tetrahedral local environment. For each of the structure models, we then calculated the corresponding RDF and an EXAFS spectrum using the *EvAX* code,^41^ which, in turn, used the *FEFF* code^42^ for ab initio EXAFS modeling. The pairs of the calculated EXAFS spectra χ_*i*_(*k*) and partial RDF sets  were then constructed, where  is a vector, consisting of four concatenated partial RDFs, corresponding to the M_Th_–O, M_Th_–M, M_Oh_–O, and M_Oh_–M bond length distributions. Here and further, M is either Fe or Co, and M_Th_ and M_Oh_ refer to tetrahedrally or octahedrally coordinated metal site, respectively. M without subscript indicates neighboring metal atom that can have either tetrahedral or octahedral coordination.

**Figure 4:**
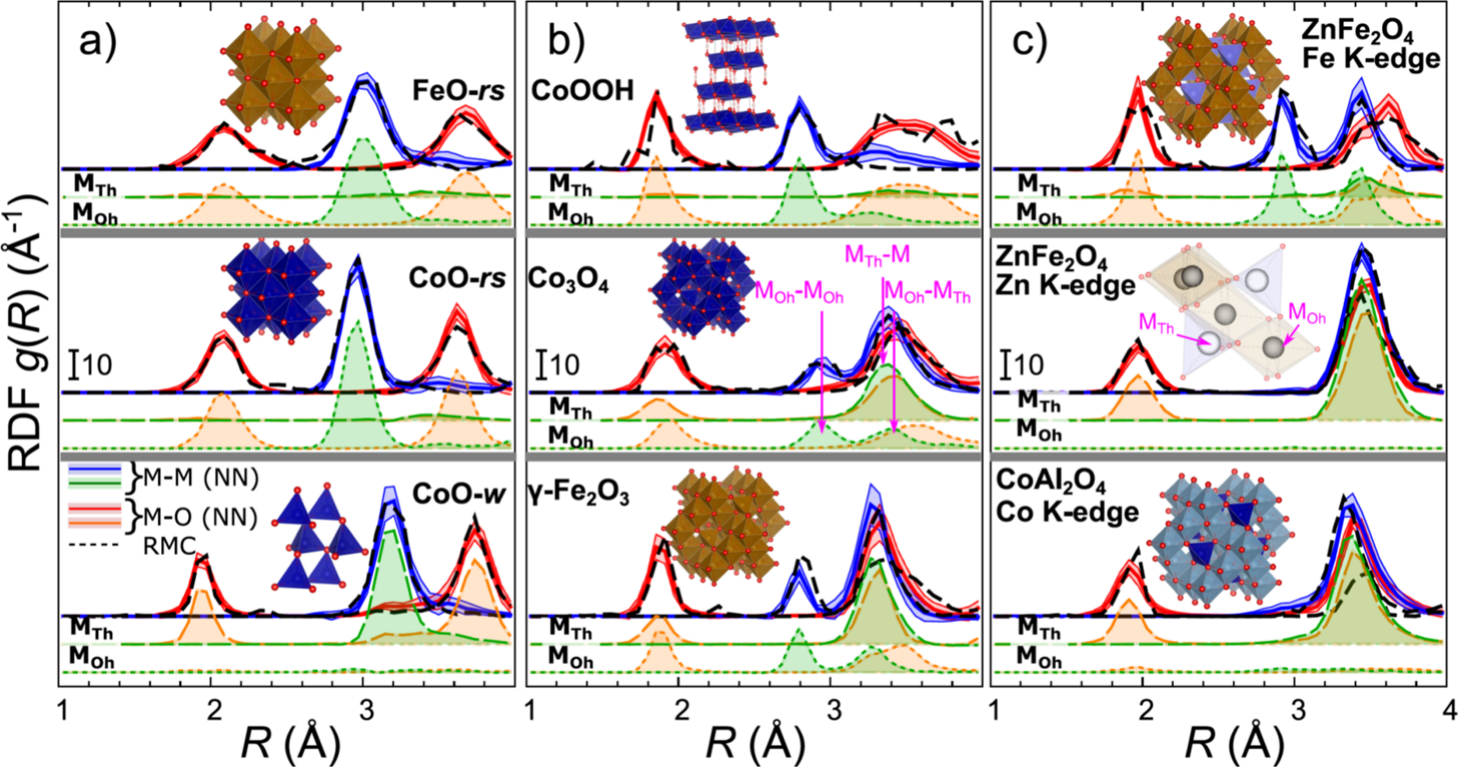
Validation of the NN-EXAFS method using RMC simulations and EXAFS spectra for reference materials (rocksalt-type CoO and FeO, wurtzite-type CoO (a), CoOOH and spinel-type Co_3_O_4_ and γ-Fe_2_O_3_ (b), and spinel-type ZnFe_2_O_4_ and CoAl_2_O_4_ (c). The total M–O and M–M RDFs, calculated from the same experimental Fe, Co, or Zn K-edge EXAFS data using RMC and NN-EXAFS methods are shown, as well as partial M_Th_–O, M_Th_–M, M_Oh_–O, and M_Oh_–M RDFs, extracted by NN-EXAFS method. The insets show the structure models used for RMC simulations, as well as a schematic depiction of tetrahedral and octahedral sites in a spinel structure. M denotes here either Fe or Co. M_Th_ and M_Oh_ refer to tetrahedrally or octahedrally coordinated metal site, respectively. The corresponding M_Th_–M, M_Oh_–M_Oh_, and M_Oh_–M_Th_ RDF peaks are indicated in (b). RDFs are shifted vertically for clarity. Blue lines correspond to the total metal–metal radial RDFs. Shaded blue areas indicate the uncertainty of the NN results. Green lines indicate the partial RDFs calculated for tetrahedrally coordinated and octahedrally coordinated metal sites. Red lines correspond to the total metal–oxygen RDFs, with the NN uncertainty indicated as red shaded areas. The orange lines correspond to the partial RDFs calculated for tetrahedrally coordinated and octahedrally coordinated metal sites. Note that since Al is not a neighbor of Co and Fe in the Periodic Table, application of our NN, trained on Fe and Co oxides exclusively, to EXAFS data interpretation in CoAl_2_O_4_ should be considered with caution, and only qualitatively correct results should be expected.

To construct model spectra and the corresponding RDFs for mixtures, we randomly pick three spectra and corresponding RDFs for pure compounds and construct their linear combination with random weights *w*_*ji*_. We repeat this process 10,000 times. The obtained set of spectra and RDFs for mixtures are then used for NN training. We emphasize that after the training on theoretical EXAFS data, the accuracy of the NN must be validated with the experimental data for well-defined reference compounds. In Figures 4 and S11, we demonstrate the results of such a validation using experimental Co K-edge, Fe K-edge, Ni K-edge, Mn K-edge, and Zn K-edge reference spectra for a broad range of reference materials. Note that while our NN is trained only on EXAFS data for Co K-edge and Fe K-edges, it can be used for the interpretation of EXAFS spectra of elements that are their neighbors in the Periodic Table. To test the accuracy of the obtained RDFs yielded by our NN, we compare them with RDFs, independently extracted from the same EXAFS spectra by another method—reverse Monte Carlo (RMC) simulations.^41,43^ The latter can be used for the interpretation of EXAFS spectra in well-defined pure compounds but not in the mixtures. Good agreement between the RDFs extracted by NN and RMC methods for reference compounds, as shown in Figures 4 and S11, gives us confidence in the accuracy of the developed method. We particularly highlight the fact that the NN correctly assigns the extracted RDFs to tetrahedral or octahedral metal species, that is, the RDF of tetrahedral sites is equal to 0 for the reference materials featuring only absorbing atoms with octahedral coordination and vice versa. This is also true for (nearly) normal spinels such as ZnFe_2_O_4_, CoAl_2_O_4_, and ZnGa_2_O_4_ (Figures 4c and S11b), where the experimental EXAFS spectra are dominated by contributions of the absorbing atoms (Zn, Fe, or Co) with only one kind of local environment. For spinel-like compounds such as Co_3_O_4_, Fe_3_O_4_, and γ-Fe_2_O_3_, featuring absorbing metal sites of both types, NN-EXAFS correctly yields non-zero contributions for all partial RDFs. The relative concentrations of tetrahedral and octahedral sites can be easily quantified from NN-EXAFS results. Here, we first note that by our definition of RDFs, CNs can be calculated from the partial RDFs by integrating the RDF peak, corresponding to a specific coordination shell. For mixtures, however, the NN-EXAFS yields RDF scaled by the concentration of given species. Therefore, the result of the RDF integration is an *apparent* CN. Apparent CNs *Ñ* are always lower than the true CNs *N*, which we define as the true number of neighbors of a given type (e.g., oxygen) for each absorbing atom with specific coordination (e.g., tetrahedral) in a pure compound: *Ñ* = *wN*, where *w* is the concentration of M_Th_ or M_Oh_. Since, by definition, the true CNs *N* for M_Th_–O and M_Oh_–O bonds in the first coordination shell are known and equal to 4 and 6, respectively, knowing the apparent CNs *Ñ* from the first RDF peak integration, we can calculate the concentrations of M_Th_ and M_Oh_ as *w* = *Ñ*/*N*. The concentrations of tetrahedrally coordinated species calculated from NN-EXAFS for reference compounds are compared with the respective true values in Figure S12.

Furthermore, we test the accuracy of the NN-EXAFS method, by applying it to analyze synthetic model spectra, where we combined experimental Co K-edge EXAFS spectra of wurtzite-type CoO (featuring only tetrahedrally coordinated Co) and CoOOH (featuring only octahedrally coordinated Co), with weights *w*_Th_ and *w*_Oh_ = 1 – *w*_Th_, respectively. Using the trained NN, we reconstructed the corresponding RDFs and estimated the *w*_Th_ values. As one can see from Figure S12, the *w*_Th_ values yielded by the NN-EXAFS method are indeed in a reasonable agreement with the true values of *w*_Th_. We observe that our method is the most accurate for systems containing nearly pure tetrahedral or pure octahedral species. For samples with intermediate concentrations of tetrahedral sites, the NN method tends to systematically overestimate the concentrations of tetrahedrally coordinated absorbing sites by ca. 10–20 percent points. Nonetheless, such an accuracy is sufficient to track the phase transitions and degree of spinel inversion in OER catalysts (which are not easily accessible by any other EXAFS interpretation approach). Furthermore, since this overestimation is systematic, we can account for it when interpreting the NN-EXAFS results. Further details of the NN construction, training, and validation are given in Note 2 in Supporting Information and Figures S11–S13.

### 3.3 Deciphering of EXAFS Data for Working Co_*x*_Fe_3–*x*_O_4_ Catalysts

**Figure 5:**
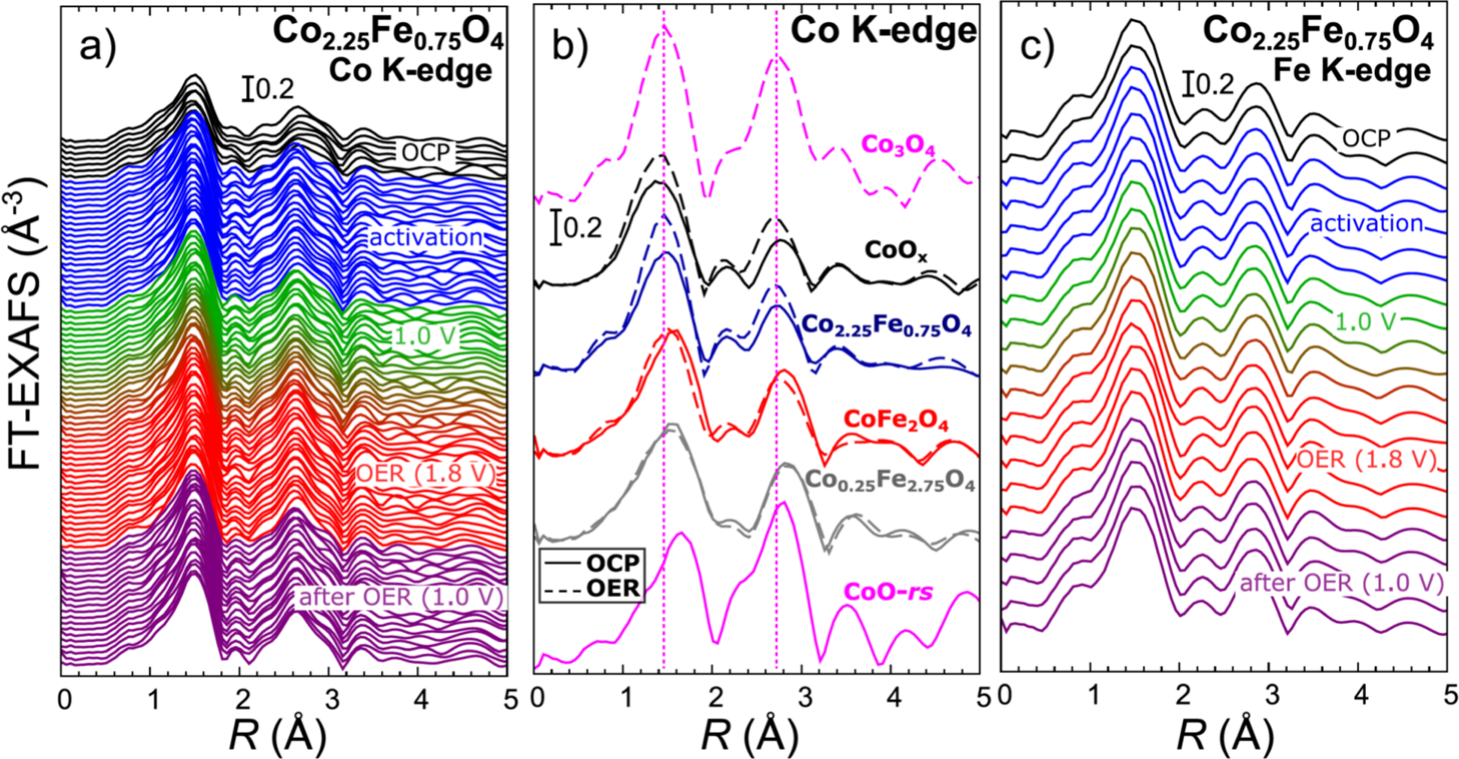
Evolution of Fourier transformed (FT) Co K-edge (a,b) and Fe K-edge (c) EXAFS spectra during the activation and under OER conditions for Co_2.25_Fe_0.75_O_4_ (a,c) and CoO_*x*_, CoFe_2_O_4_, and Co_0.25_Fe_2.75_O_4_ (b) nanocatalysts. Spectra are shifted vertically for clarity. A comparison of Fourier-transformed Co K-edge EXAFS spectra for all Co_*x*_Fe_3–*x*_O_4_ samples with different Co to Fe ratio is shown in (b). The spectra collected for the as-prepared samples under OCP and for the samples under OER conditions are shown, as well as the spectra for reference oxides CoO (rocksalt structure) and Co_3_O_4_ (spinel structure). In all cases, FT was carried out in the *k*-range between 1 and 9 Å^–1^. In (a,b), each depicted spectrum is obtained after averaging the QXAFS data collected within 50 s, except for the Co_0.25_Fe_2.75_O_4_ sample in (b). For the latter, as well as for Fe K-edge spectra in (c), each spectrum was averaged within 200 s due to the low loading of the respective metals.

Figures 5a,b and S14 and S15 show the evolution of the Co K-edge EXAFS for our Co_*x*_Fe_3–*x*_O_4_ catalysts during the activation and under OER conditions. Fourier-transformed EXAFS data are shown in Figures 5 and S15, while the raw EXAFS data in *k*-space are shown in Figure S14. In all cases, Co K-edge FT-EXAFS spectra are dominated by two broad asymmetric peaks. The first one located between 1 and 2 Å (phase uncorrected) corresponds to the contribution of all Co–O bonds in the first coordination shell. The second peak between ca. 2.5 and 3.0 Å, in turn, is dominated by various Co–M contributions, including the bonds between octahedrally coordinated sites (Co_Oh_–M_Oh_), tetrahedrally coordinated sites (Co_Th_–M_Th_), mixed bonds between octahedral and tetrahedral sites (Co_Oh_–M_Th_ and Co_Th_–M_Oh_), and the bonds between metal sites in non-spinel phases. Note that the Co_Oh_–M_Oh_ bond is expected to be significantly shorter (by ca. 0.7 Å) than other metal–metal bonds (see Figure 4). The length of the interatomic bonds is also affected by the metal oxidation state. In general, higher metal oxidation state results in shorter interatomic distances. Therefore, in rocksalt-type oxides, the Co_Oh_–M_Oh_ bond is slightly longer than the Co_Oh_–M_Oh_ bond in spinel structures. The interplay between these contributions results in a complex shape of the second FT-EXAFS peak for our catalysts.

The FT-EXAFS peaks shift systematically toward larger interatomic distances upon an increase in Fe concentrations (Figure 5b). For the CoO_*x*_ sample, the positions of the main FT-EXAFS peaks resemble those of the Co_3_O_4_ reference. The position of the Co–O peak in the Co-poor samples, in turn, resembles that in the CoO-*rs* reference. The differences in the Co–O position thus could reflect the progressively lower Co-oxidation state upon an increase in the Fe concentration, in agreement with the observations from XANES data. Note, nonetheless, that the Co–M peak in our Co-poor catalysts is at higher interatomic distances than the Co–Co peak in CoO-*rs*. Therefore, not only the differences in the Co-oxidation state but also in crystallographic structures need to be considered when interpreting the positions of FT-EXAFS features.

One should note here that when discussing EXAFS spectra in materials featuring elements that are neighbors in the Periodic Table, especially if the concentration of the lighter element (Fe in our case) is much higher than that of the heavier element (Co in our case), “leaking” of EXAFS oscillations of the lighter element into the EXAFS spectrum of heavier element can sometimes be observed.^44^ This could result in distortions of Co K-edge EXAFS spectra for Co-poor materials. In Supporting Information Note 3 and Figure S16, we demonstrated that distortions are expected to be small for the Co_0.25_Fe_2.75_O_4_ sample and completely negligible for samples with higher Co to Fe ratio.

During the activation, the intensity of both main FT-EXAFS peaks increases for Co-rich catalysts (CoO_*x*_ and Co_2.25_Fe_0.75_O_4_), while the changes in EXAFS spectra for Co-poor samples are more subtle. These observations are in agreement with the trends in the Co K-edge XANES data. The increase in the FT-EXAFS peak amplitudes could be attributed either to an increase of the fraction of Co species with higher CN (i.e., octahedrally coordinated), or to a disorder effect, namely, lower disorder in the activated samples due to a more homogeneous structure. Quantitative analysis is needed to tell apart the roles of these two effects.

**Figure 6:**
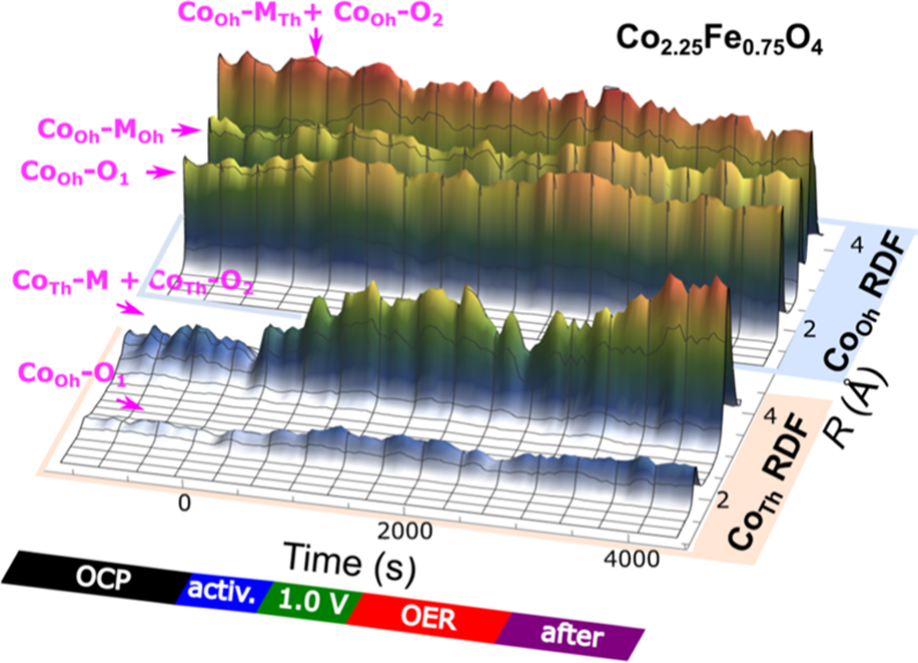
Evolution of RDFs for tetrahedrally and octahedrally coordinated Co sites in Co_2.25_Fe_0.75_O_4_ during the activation and under OER conditions at 1.8 V_RHE_. For both sites, the total RDFs (a sum of Co–O and Co–M partial RDFs) are shown.

**Figure 7:**
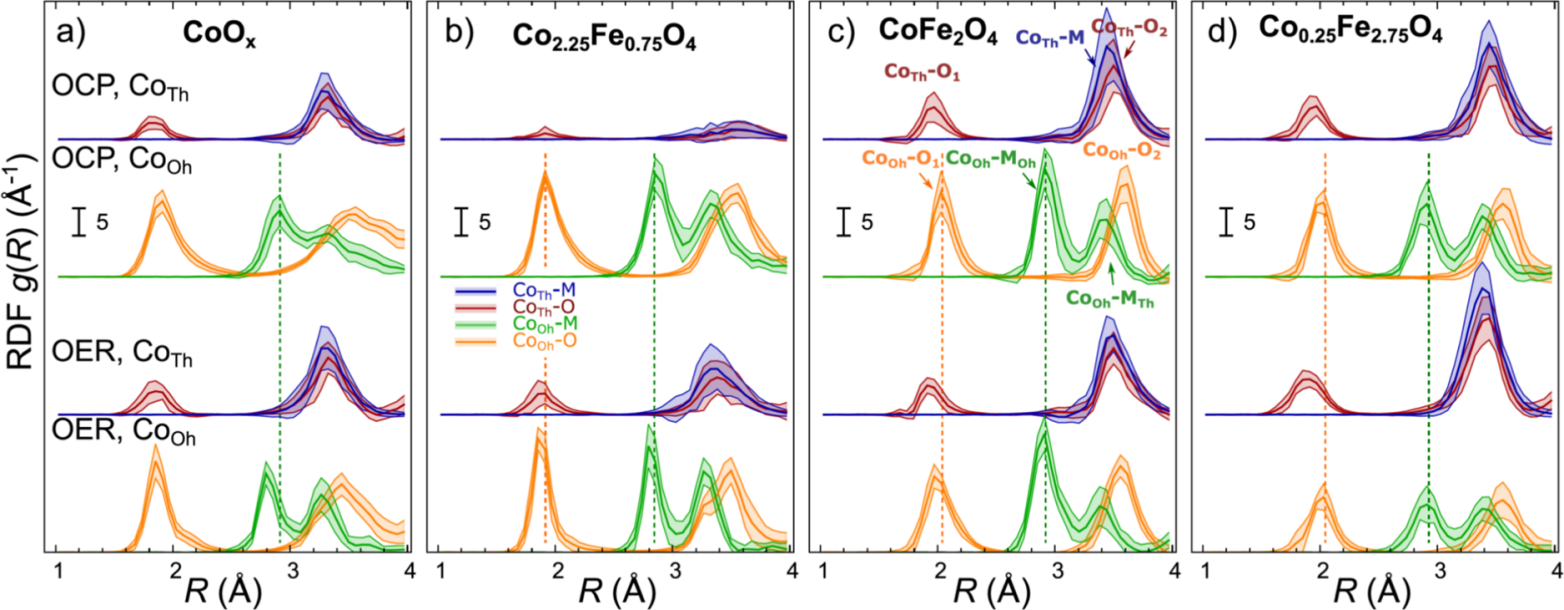
Partial RDFs extracted by NN from *operando* Co K-edge EXAFS data for CoO_*x*_ (a), Co_2.25_Fe_0.75_O_4_ (b), CoFe_2_O_4_ (c), and Co_0.25_Fe_2.75_O_4_ (d). The plotted RDFs correspond to Co–O and Co–M contributions for tetrahedrally and octahedrally coordinated Co sites for the as-prepared samples under OCP conditions and for samples under OER conditions at 1.8 V. RDFs are shifted vertically for clarity.

**Figure 8:**
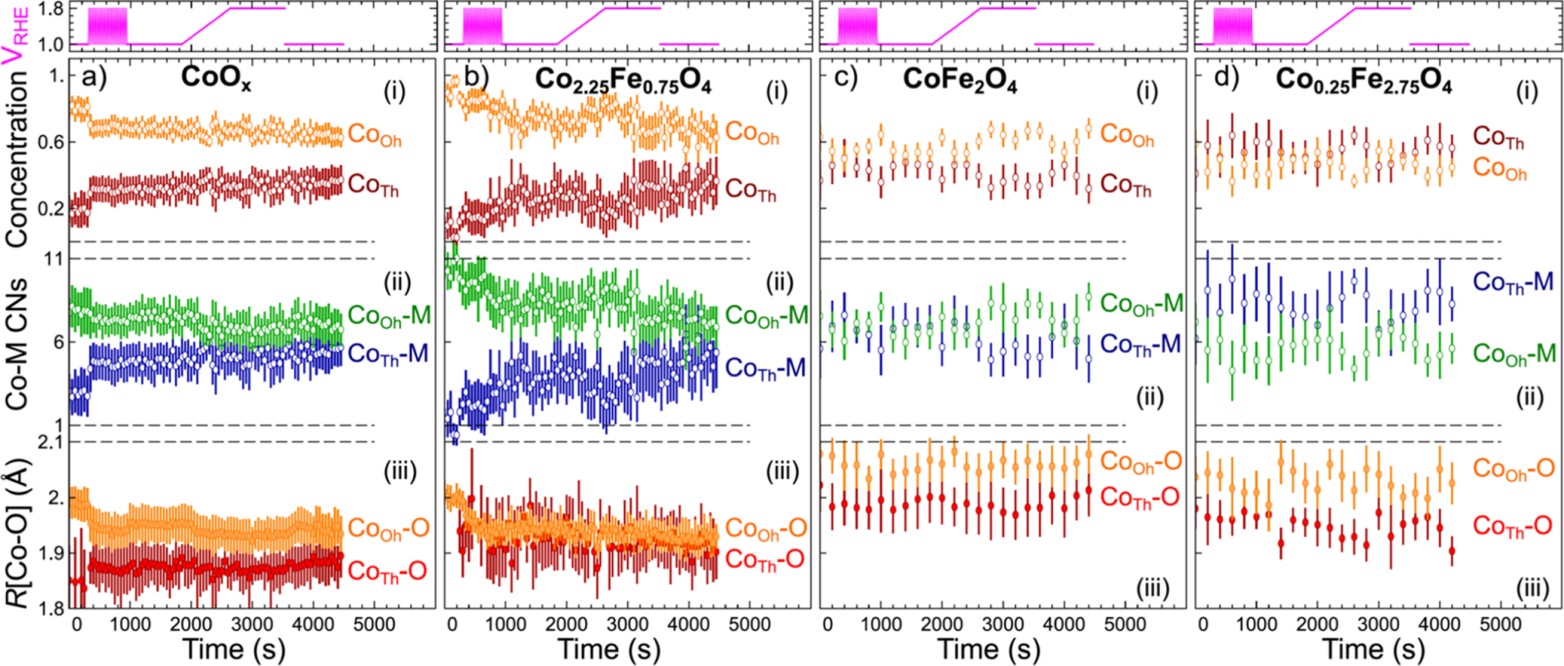
Evolution of the Co local structure parameters, extracted by integrating the RDFs yielded by the NN-EXAFS analysis approach. Structure parameters obtained from EXAFS spectra collected during activation and under OER conditions (1.8 V_RHE_) for CoO_*x*_ (a), Co_2.25_Fe_0.75_O_4_ (b), CoFe_2_O_4_ (c), and Co_0.25_Fe_2.75_O_4_ catalysts (d) are shown. Depicted quantities are the (i) concentrations of Co_Th_ and Co_Oh_ sites, obtained from the first shell apparent CNs as /4 and /6, respectively; (ii) total Co_Th_–M and Co_Oh_–M apparent CNs, obtained by integrating the Co_Th_–M and Co_Oh_–M partial RDFs in the *R*-range between 1 and 4 Å; and (iii) average Co_Th_–O and Co_Oh_–O interatomic distances, obtained by integrating the Co_Th_–O and Co_Oh_–O partial RDFs in the *R*-range between 1 and 2.56 Å.

The complex, asymmetric shape of the FT-EXAFS peaks that reflects the underlying broad, multimodal bond-length distributions limits the applicability of conventional EXAFS fitting approaches. Nonetheless, the quantitative analysis of these transformations is possible by using the NN-EXAFS approach. In Figure 6, we show an example of the NN-EXAFS analysis results, obtained for the Co_2.25_Fe_0.75_O_4_ sample. We observe a clearly different evolution of the RDFs for the tetrahedrally and octahedrally coordinated Co sites during the activation, under OER conditions and after the reaction. A detailed comparison of the selected partial RDFs for all Co_*x*_Fe_*y*_O_*z*_ samples is shown in Figure 7. Furthermore, for a more quantitative comparison, we integrate the RDF peaks to obtain CNs and average interatomic distances. In particular, we have calculated the apparent first shell (Co–O) CN both, for the tetrahedrally and octahedrally coordinated Co species, and used it to estimate the concentration of the corresponding Co species (Co_Th_ or Co_Oh_). The results are shown in Figure 8. The apparent total Co-M CNs for Co_Th_ and Co_Oh_ and the changes in the average Co–O interatomic distance are also shown in Figure 8. The RDFs for all samples are dominated by a spinel-like contribution, with a characteristic overlap of Co_Th_–M and Co_Th_–O_2_ peaks in the partial RDFs for Co_Th_ species and distinct Co_Oh_–M_Oh_ and Co_Oh_–M_Th_ peaks in the partial RDF for Co_Oh_ species (Figure 7). We note nonetheless, that for the Co-rich samples, especially for the CoO_*x*_ before the activation, the Co_Oh_–O_2_ peak is noticeably broader than that for the other samples, and that the Co_Oh_–M_Oh_ and Co_Oh_–M_Th_ peaks are less separated. The additional intensity in-between those peaks can be attributed to the contribution of different octahedrally coordinated Co species with longer interatomic distances, which implies lower Co-oxidation state. This strongly suggests the presence of Co(II) species in the octahedral (rocksalt-like) environment. This latter contribution is decreased during the activation.

Overall, for the CoO_*x*_ sample, we observe that the majority of Co species (ca. 80%) are octahedrally coordinated, with a relatively long (ca. 2.0 Å) average Co–O bond length. This also points toward a strong contribution of Co(II) species in rocksalt-like environment, for which such a long Co–O bond is typical (see Figure 2d). Upon activation, the contribution of Co_Oh_ species decreases, while the contribution of tetrahedrally coordinated Co species increases, until the Co_Oh_ to Co_Th_ ratio becomes close to the 2:1 ratio typical for the Co_3_O_4_ spinel structure (Figure 8a). These changes are paralleled by a decrease in both Co–O and Co–Co distances for the Co_Oh_ species. This indicates the conversion of the rocksalt-like Co environment phase into a spinel-like structures with a higher average oxidation state for Co_Oh_ species. We also note that the decrease of the Co_Oh_–O bond length during the activation and later under OER conditions coincides with a slight decrease in the total Co_Oh_–M CN. This could be interpreted as the formation of a layered CoOOH-like structure (in addition to the spinel-like phase), for which the total Co_Oh_–M CN (Co_Oh_–M_Oh_ + Co_Oh_–M_Th_) is lower (6 vs 12 for Co_Oh_ in spinel or rocksalt-type structure). We observed that under OER, the true Co_Oh_–M CN *N*, corrected for the concentration of octahedrally coordinated species (), decreases to ca. 10, indicating that approximately one-third of the octahedrally coordinated species forms a CoOOH-like structure. Unlike the conversion of the rocksalt-like Co environment into spinel-like structures, the further Co-oxidation and formation of CoOOH-like structure under OER conditions are reversible, as evidenced by the increase in the Co_Oh_–M CN and in the Co_Oh_–O bond length after the potential is returned to the resting value of 1.0 V_RHE_.

For Co_2.25_Fe_0.75_O_4_, we also observe strong changes in the Co local environment under electrochemical treatment. As shown in Figures 6, 7b, and 8b, the contribution of Co_Th_ sites is low for the as-prepared Co_2.25_Fe_0.75_O_4_ sample, which we attribute both, to the presence of the rocksalt-like structures, and to the fact that in the spinel phase, due to the high Co loading in this sample, cobalt is forced to occupy more abundant octahedral sites. However, during the activation, the Co_Th_ contribution grows significantly, while the Co_Oh_–O bond decreases, suggesting an irreversible transformation of the rocksalt-like structures into a spinel-like motifs with Co_Oh_ to Co_Th_ ratio similar to that in Co_3_O_4_. Unlike it was in the case for the CoO_*x*_ catalyst, we observed that the contribution of Co_Th_ is transiently decreased again, when the sample is exposed to OER conditions. This could imply that Co reversibly replaces Fe in some of the octahedral spinel sites. However, the lack of pronounced changes in the Fe K-edge XAFS data speaks against this hypothesis. In fact, for this Fe-poor sample, any changes in the Co_*x*_Fe_3–*x*_O_4_ spinel phase should be much better visible from the Fe perspective. The lack of such changes in the Fe XAFS suggests that the structural transformations in the local structure of Co are again more likely to be related to the formation of edge-sharing Co^3+^–O_6_ octahedral units, which are presumably the active sites for the OER. We also note here that the changes in the apparent Co_Oh_–M and Co_Th_–M CN for this sample are merely proportional to the changes in the concentrations of Co_Oh_ and Co_Th_ species. The edge-sharing Co^3+^–O_6_ octahedra thus form here more three-dimensional, likely strongly disordered and defective arrangement, rather than well-defined layered CoOOH-like phase with reduced Co_Oh_–M CNs. Assuming that the observed change in the concentrations of tetrahedral and octahedral species (ca. 10 percent points) under OER is solely due to the formation of Co^3+^–O_6_, we estimate that under working conditions ca. 10% of Co atoms are involved in the formation of these species.

In the CoFe_2_O_4_ sample (Figures 7c and 8c), we do not observe a large contribution of the rocksalt-like structures, and the catalyst appears to be spinel-like already in the as-prepared state. Despite higher Fe concentration, the ratio of Co_Oh_ to Co_Th_ sites is similar to that in the previous cases, suggesting that the distribution of Co over octahedral and tetrahedral sites is close to random, with no strong preferences for Co to occupy tetrahedral sites. We observe that Co–O bonds in this sample are noticeably longer than that for the more Co-rich samples, suggesting lower average Co-oxidation state, and thus, showing the effect of Fe on the Co valence. Regarding the behavior of this sample under OER conditions, we note that, similar to the Co_2.25_Fe_0.75_O_4_ sample, the contribution of Co_Oh_ sites reversibly increases under OER conditions (again, by ca. 10 percent points), suggesting the formation of similar Co^3+^–O_6_ clusters.

For the Co_0.25_Fe_2.75_O_4_ sample (Figures 7d and 8d), we observe similar structure to CoFe_2_O_4_, but the contribution of the Co_Th_ sites appears to be slightly higher, suggesting thus a lower degree of inversion in the spinel structure, that is, in this sample low-valent Co species do show a preference toward occupying tetrahedrally coordinated sites. For this sample, and within the uncertainty of our analysis, we did not detect any significant variations in the local structure for this sample during the electrochemical treatment, except a slight continuous decrease in the Co–O bond length, which agrees with the gradual oxidation of Co in this sample evidenced by XANES. This is in clear contrast to the transformations observed for the rest of the samples with higher Co loading.

Finally, we discuss the NN-based analysis of the Fe K-edge EXAFS. Corresponding EXAFS spectra are shown in Figures 5c and S17 and S18. All EXAFS spectra strongly resemble the spectra for the γ-Fe_2_O_3_ spinel. The positions of the main FT-EXAFS peaks in Fe_3_O_4_ are also similar, but the peak intensities in γ-Fe_2_O_3_ match better our observations for the Co_*x*_Fe_3–*x*_O_4_ catalysts (Figure S18). In agreement with our XANES data, only very subtle changes in the Fe K-edge EXAFS can be observed during the electrochemical treatment, the largest being the modifications in the contributions of distant coordination shells for the Co_2.25_Fe_0.75_O_4_ sample. Unlike it was for Co K-edge EXAFS, the Fe K-edge EXAFS spectra for different samples do not exhibit significant changes in the positions of the main FT-EXAFS peaks upon the changes in the Co to Fe ratio (Figure S19).

The partial RDFs, extracted from Fe K-edge EXAFS, are shown in Figure S20. Figure S21 shows the evolution of the Fe local structure parameters, as obtained by the integration of the partial RDFs. The most interesting effect appears to be the evolution of the Co_2.25_Fe_0.75_O_4_ sample, where we observe a larger initial concentration of Fe_Oh_ species, which get irreversibly partially transformed into Fe_Th_ species during the activation (the concentration of Fe_Th_ increases by ca. 20 percent points). Thus, the evolution of Fe species in this sample is similar to that of Co species and suggests the presence of a non-spinel iron oxide in the initial structure, which gets transformed into spinel during the activation. We note here that no significant changes in the Fe–O bond lengths are observed during this process, suggesting that the decrease in the Fe-oxidation state in this process is small, and, thus, the initial oxide phase cannot be directly associated with the rocksalt-like Fe^2+^ phase (wüstite), but, perhaps, can be rather associated with the incorporation of Fe^3+^ into a defective CoO-like environment.

For more Fe-rich samples, non-spinel-like structures are not observed, and no significant transformations in the Fe local structure can be detected by our NN-EXAFS approach. Both, for CoFe_2_O_4_ and Co_0.25_Fe_2.75_O_4_, we observe a higher concentration of the Fe_Th_ species than that for Co_2.25_Fe_0.75_O_4_, which is in agreement with the conclusions from the XANES data analysis. In fact, from NN-EXAFS, the contribution of Fe_Th_ for the Fe-rich samples is even larger than that of Fe_Oh_. This is not consistent with an Fe-rich spinel structure, and we attribute this result to the systematic overestimation of the Fe_Th_ concentrations by our NN-EXAFS, as we observed in Figure S12. Indeed, the concentrations of Fe_Th_ and Fe_Oh_ that NN-EXAFS yields for CoFe_2_O_4_ and Co_0.25_Fe_2.75_O_4_ catalysts are similar to those yielded for Fe_3_O_4_ and γ-Fe_2_O_3_ references (Figure S12).

## 4 Discussion

**Figure 9:**
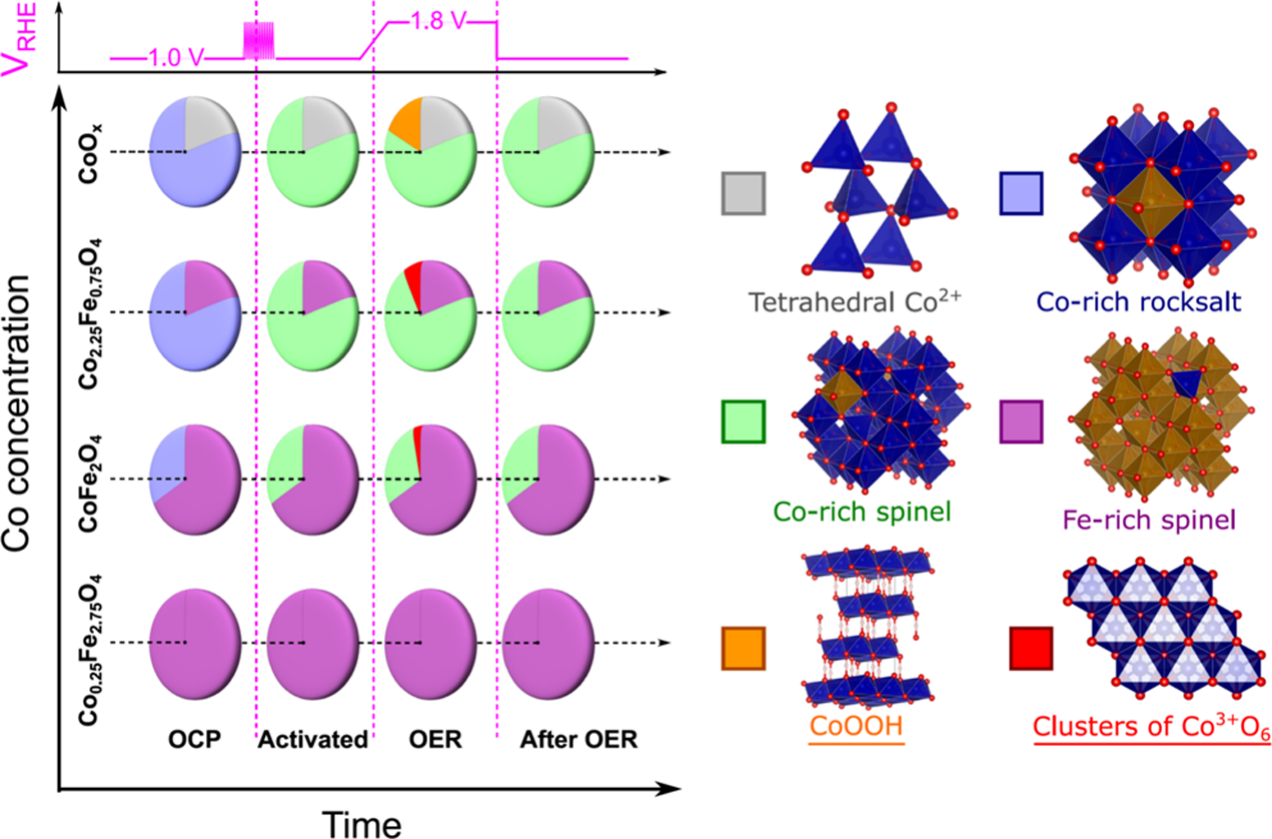
Schematic depiction of the structure and composition-dependent transformations of the Co_*x*_Fe_3–*x*_O_4_ electrocatalyst after activation (at 1.0 V_RHE_) and during the OER (at 1.8 V_RHE_) in 0.1 M KOH electrolyte. Red and orange sectors show active species for the OER. The structure of the Co_0.25_Fe_2.75_O_4_ sample is assigned to a single Fe-rich spinel-like phase containing both Fe and Co species. For the CoFe_2_O_4_, the concentration of the Fe-rich phase (purple sector) is assumed to be equal to the Fe to Co ratio in this sample. For the Co_2.25_Fe_0.75_O_4_ sample, the concentration of the Fe-rich species is reduced by 20% to account for the incorporation of Fe into Co-rich structures, as deduced from the NN-EXAFS analysis at Fe K-edge. These 20% are added to the concentration of Co-rich rocksalt-like (blue sectors) and spinel-like (green sectors) species. The relative concentrations of distinct Co-rich phases for CoO_*x*_, Co_2.25_Fe_0.75_O_4_, and CoFe_2_O_4_ are deduced from the NN-EXAFS analysis at the Co K-edge.

The machine-learning-based analysis of our XANES and EXAFS data, collected at the K-absorption edges of Co and Fe, provides valuable insights into the dependency of the local structure and oxidation state of the Co_*x*_Fe_3–*x*_O_4_ nanocatalysts on the Co to Fe ratio, and into the evolution of these catalysts during the electrochemical activation and under OER conditions. We highlight that the coexistence of different structures and the segregation of Fe and Co species play key roles in the observed changes in the experimental spectra (Figure 9). We found that the results of advanced XANES and EXAFS data analyses are in a good agreement, but only through their complementary application, a complete picture of the evolution of these heterogeneous materials can be obtained.

For the pure CoO_*x*_ catalyst, both XANES and EXAFS data provide compelling evidence that at least four different Co species are present in this sample and evolve under electrochemical treatment. In agreement with the ex situ XRD data, we observe that the as-prepared samples contain a large fraction of rocksalt-like CoO species. During the activation treatment, this oxide is irreversibly transformed into a spinel-like structure whose local structure resembles that of Co_3_O_4_. The latter, in turn, is reversibly oxidized into a CoOOH-like state, resulting in a contraction of all interatomic distances around Co_Oh_ sites. The evolution of the Co_Oh_ sites in the CoO_*x*_ catalyst thus matches well the unified picture of the transformations of cobalt oxides under OER conditions proposed by Bergmann et al. in ref (9), where the edge-sharing Co^3+^–O_6_ octahedra are shown to be the main active site for the OER. In addition to the rocksalt-like species, the as-prepared CoO_*x*_ catalyst features also another type of oxide structures. XRD identified this phase as corresponding to wurtzite-type CoO.^14^ From XAFS, while the presence of tetrahedrally coordinated Co^2+^ is confirmed in the as-prepared samples, we do not see clear evidences of the presence of wurtzite-type CoO. According to our NN-EXAFS analysis, the local environment of these tetrahedral Co^2+^ sites resembles more that of the disordered spinel-like structure. Likely, the sample features a broad range of different disordered oxides. Different experimental techniques have different sensitivities to the different components of this mixture. Nonetheless, based on our PCA-XANES, we can conclude that these particular tetrahedrally coordinated Co^2+^ species are not characteristic for the bimetallic Co_*x*_Fe_*y*_O_*z*_ catalysts discussed below. Furthermore, the tetrahedrally coordinated Co species in the CoO_*x*_ catalyst seem to be much less electrochemically active than the octahedrally coordinated ones, as suggested by the lack of changes in the Co–O distances in the RDFs for Co_Th_ in these samples, as extracted by the NN-EXAFS method.

The interpretation of the results obtained for Co_*x*_Fe_3–*x*_O_4_ catalysts is more intricate. First, we need to reconcile the observation that the Fe K-edge XAFS data for all samples are very similar and exhibit relatively minor changes under electrochemical treatment, while the Co K-edge XAFS data show pronounced changes in the local environment and oxidation state of Co species. To explain this contradiction, we must conclude that Fe and Co species are strongly segregated. Our analysis suggests that irrespective of the Fe to Co ratio, in all of our samples Fe is nearly completely in a 3+ oxidation state and exhibits spinel-like local environment that features both tetrahedrally and octahedrally coordinates sites. We thus conclude that in all our samples, the majority of Fe species segregate to form γ-Fe_2_O_3_-like structures. Some small amounts of Co^2+^ can be incorporated into this structure, forming Co-poor Co_*x*_Fe_3–*x*_O_4_ spinel-like structures. We expect that in our sample with the lowest Co content (Co_0.25_Fe_2.75_O_4_), this structural motif is present in its purest form. The distribution of Co incorporations over tetrahedral and octahedral sites appears to be nearly random in this structure, although some slight preference for tetrahedral sites can be detected in the Co K-edge EXAFS data for the Co_0.25_Fe_2.75_O_4_ sample. Importantly, these Fe-rich spinel-like species appear to be electrochemically passive since no response of either Fe or Co species to changes in the applied potential can be detected, and samples containing the largest fraction of this species are also the least OER active.

For samples with higher Co loadings, the Fe-rich structures coexist with segregated Co-rich structures. The latter also might include some small amounts of Fe species incorporated, resulting in different local structures and electrochemical properties than those observed in the pure CoO_*x*_ sample. In the as-prepared samples at OCP, this Co-rich phase is dominated by a rocksalt-like structure (featuring also Fe inclusions, as we deduced from the Fe K-edge EXAFS data and also ex situ XRD). The increased Co-oxidation state for samples with higher Co loadings, observed both in Co K-edge XANES and EXAFS data, suggests that some Co species in higher than 2+ state (e.g., Co_3_O_4_-like species) must also contribute to the Co-rich structures. Unlike the Fe-rich species, the Co-rich species are actively responding to the changes in the electrochemical conditions. During the activation treatment, the rocksalt-like structures are first irreversibly converted into the spinel-like motifs. Furthermore, during the activation treatment and under OER conditions, this structure is further oxidized. We highlight here that this results in an increase in the oxidation state both, for Co and the minority Fe species, and contraction of interatomic distances. The contribution of tetrahedrally coordinated Co species is decreased during this process, suggesting the formation of clusters of edge-sharing Co^3+^–O_6_ octahedra. The latter are believed to be the active species for the OER.^8,45^ However, the results, obtained in this work, also suggest that the incorporation of small amount of Fe into Co-rich phases also plays an important role in enhancing the OER activity. This study thus contributes to resolving the debate, whether Fe is directly involved in the catalytic mechanisms, as suggested by other recent studies, or if the FeO_*x*_ phases might constitute a simple structural stabilizer of the active CoO_*x*_ components.^4,6,20,46,47^ Both scenarios could explain the higher OER activity for Co_2.25_Fe_0.75_O_4_ than that for the CoO_*x*_ sample. Our results showing that Fe species in Co-rich structures are also getting further oxidized under OER conditions, suggest that Fe plays an active role in the reaction mechanism.

In summary, our study shows that the majority of Fe and Co species are strongly segregated, forming spinel-like Fe-rich structures and several distinct Co-rich structures, respectively. The latter are gradually oxidized under the activation treatment and OER conditions. Nonetheless, the interactions between Fe and Co, such as the incorporation of a small amount of Co into Fe-rich structures and vice versa, also play an important role. We observe that the evolution of Co-rich structures in working bimetallic oxides is different from that in our pure CoO_*x*_ catalysts. In the former case, the formation of disordered, compact clusters of Co^3+^–O_6_ octahedra as the main active sites is observed under OER conditions. In the CoO_*x*_ catalyst, in turn, our results indicate the formation of a layered structure resembling that of CoOOH. At the same time, while for the Co-poor catalyst the Fe species remain electrochemically passive, for more Co-rich catalysts our results clearly reveal further oxidation of the Fe species under OER conditions, suggesting that Fe can play a direct role in the OER mechanism.

## 5 Conclusions

This work highlights the challenges in understanding the structural changes taking place in working oxide catalysts for the OER. We demonstrate that significant transformations in the catalyst structure and chemical state are often only reflected by a subtle response in the spectroscopic data. At the same time, the structure of these electrocatalysts is quite complex and heterogeneous, and, hence, cannot be represented by a single 3D structural model. Moreover, the oxide catalysts for the OER feature both, catalytically passive spectators as well as species that are directly involved in the catalytic mechanisms. We argue that the complexity of this problem cannot be adequately addressed by conventional approaches to XAFS data analysis, such as linear combination fitting of XANES spectra and least-squares fitting of EXAFS spectra. Fortunately, as we demonstrate in this work, a possible solution is provided by machine learning methods.

In this study, we show that changes in the catalyst structure and chemical state can be resolved with a resolution of a few seconds via QXAFS measurements combined with unsupervised XANES data analysis methods such as PCA. As it is clear from this work, the oxidation processes and structural transformations in our catalysts during the activation treatment and under working conditions happen on a time scale of a few minutes, thus requiring experimental spectroscopic measurements with the corresponding high temporal resolution. On the other hand, it should be noted that these data-driven methods only work if the number of available spectra, capturing different states of the catalyst, significantly exceeds the number of spectroscopically distinct species present in the sample. For complex systems such as transition-metal oxide catalysts for the OER, this means that dozens and even hundreds of spectra are needed to accurately reveal the catalyst composition during the different stages of an electrochemical process. The application of PCA-XANES to large data sets available from the QXAFS measurements is thus a powerful approach for revealing the evolution of complex functional materials. In the present case, QXAFS combined with PCA-XANES data analysis allowed us to identify several consequent transformations in the Co chemical state during the activation of oxide catalysts. Furthermore, a particularly important result from our in-depth PCA analysis, that brings light to prior controversies in the literature,^6,47^ is that the Fe species in FeCo mixed oxides also respond to the electrochemical process and face oxidation under OER conditions. Thus, they cannot be ruled out as possible contributor to the OER mechanisms. The fact that this process was revealed by PCA-XANES, despite very small changes in the Fe K-edge XANES, highlights the high sensitivity of this approach.

Complementing the PCA-XANES data analysis, a supervised machine learning approach for EXAFS data interpretation also provides new possibilities for disentangling the evolution of different structures in working oxide catalysts. In particular, here we demonstrated that quantitative tracking of transformations of the local structure around the active metal sites is possible by using an artificial neural network method. The NN-EXAFS approach simultaneously addresses two problems of EXAFS data interpretation in complex oxides: the need to account for complex multimodal bond length distributions and the need to decipher contributions of distant coordination shells that are more sensitive to the differences between different oxide structures. Furthermore, we show that NN provides unique possibilities for tracking independently the evolution of crystallographically non-equivalent sites, thus making EXAFS analysis not only element-specific but also site-specific, which is an important advantage for understanding complex oxide materials. In particular, in this work, the NN-EXAFS approach allowed us to probe separately the response of tetrahedrally and octahedrally coordinated Co and Fe sites. We also highlight that the NN-EXAFS approach is well suited for the interpretation of QXAFS data. Indeed, after the NN training is completed, the analysis of each EXAFS spectrum can be carried out within seconds by the NN method. Therefore, the large data sets, collected in QXAFS experiments, can be efficiently and systematically processed.

Together with the results from PCA-XANES, NN-EXAFS reveals the strongly segregated structure of Co_*x*_Fe_3–*x*_O_4_ catalysts, where an Fe-rich but electrochemically passive phase coexists with the active Co-rich phase. For the latter, NN-EXAFS shows the reversible formation of edge-sharing Co^3+^–O_6_ octahedra, that have been long proposed as the main active sites for the OER. However, our new results also reveal the differences in the evolution of CoO_*x*_ and bimetallic Co_*x*_Fe_3–*x*_O_4_ catalysts. For the former, we find that in the transformations to the active layered CoOOH-like structures, mostly octahedrally coordinated Co sites are involved. At the same time, for the bimetallic spinel samples, we also observe the conversion of tetrahedrally coordinated Co sites, resulting in defective non-layered arrangements of Co^3+^–O_6_ octahedra.

Overall, this study demonstrates the great potential of machine learning methods for understanding the chemical and structural evolution of transition-metal oxide catalysts for the OER and their transformations under reaction conditions. New insights into these complex materials will be instrumental for revealing the mechanisms behind their excellent activity for the OER in alkaline electrolytes and will thus enable new pathways for the rational design of the next generation of water-splitting catalysts.

## Supporting Information Available

Details of PCA, neural network training and validation, analysis of Fe K-edge EXAFS effect on Co K-edge EXAFS, Co K-edge and Fe K-edge XANES spectra for our catalysts, reference materials and their PCA, results of neural network method validation, EXAFS fits for reference materials obtained by using RMC simulations, raw Co K-edge and Fe K-edge EXAFS data for our nanocatalysts and their Fourier transforms, and evolution of partial RDFs and local structure parameters, extracted from EXAFS spectra by the neural network (PDF)
