# Supplementary material for: Deciphering the Structural and Chemical Transformations of Oxide Catalysts during Oxygen Evolution Reaction Using Quick X-ray Absorption Spectroscopy and Machine Learning
Source: J Am Chem Soc. 2023 Feb 10;145(7):4065–80. doi: 10.1021/jacs.2c11824 (PMC9951215; doi:10.1021/jacs.2c11824)
Supplement: Supplementary file 1 — ja2c11824_si_001.pdf [file ja2c11824_si_001.pdf]

Supporting Information for

Deciphering the structural and chemical transformations of oxide catalysts during oxygen evolution reaction using quick X-ray absorption spectroscopy and machine learning

Janis Timoshenko[a]*, Felix T. Haase[a], Sascha Saddeler[b], Martina Rüscher[a], Hyo Sang Jeon[a], Antonia Herzog[a], Uta Hejral[a], Arno Bergmann[a], Stephan Schulz[b], Beatriz Roldan Cuenya[a]*

[a] Department of Interface Science, Fritz-Haber Institute of the Max-Planck Society, 14195 Berlin, Germany

[b] Institute of Inorganic Chemistry, Center for Nanointegration Duisburg-Essen (CENIDE), University of Duisburg-Essen, 45117 Essen, Germany.

*Email: janis@fhi-berlin.mpg.de, roldan@fhi-berlin.mpg.de

Supplementary Note 1: Principal component analysis of XANES data

The objective of principal component analysis (PCA) is to express the experimental XANES spectra in a dataset as linear combinations of a few linearly independent vectors (principal components) $p_j(E)$:

$$\mu_i(E) - \bar{\mu}(E) = \sum_{j=1}^{d} a_{ij} p_j(E) \tag{S1}$$

For convenience, we follow here a common practice in PCA (that, however, is not always observed in PCA-XANES analysis), and subtract from the experimental spectra $\mu_i(E)$ the averaged spectrum $\bar{\mu}(E)$, calculated over the entire dataset.[1, 2] One can notice the analogy between PCA and the linear combination analysis (LCA), commonly used for XANES data interpretation. In LCA, an experimentally measured XANES spectrum is expressed as a linear combination of spectra for a few reference compounds. The important difference between LCA and PCA is that in the latter the principal components (PCs) used for linear combinations are abstract vectors that do not correspond to any particular pure species. On the other hand, since PCs are deduced from the experimental dataset itself using linear algebra methods, PCA works even in the cases where the lack of sets of suitable reference spectra prevents the application of LCA. In particular, an important objective of PCA is to determine the number of principal components *d* that is needed to reproduce all the meaningful spectroscopic variations in the experimental dataset. This number defines the dimensionality of the dataset, and is thus related to the number of spectroscopically distinct species present in the course of the reaction. Note that the actual number of species in the sample may be larger, if the spectra for two or more distinct species are very close, or if the concentrations of two species are proportional to each other at all times during the experiment.

The principal components $p_j(E)$ and the corresponding projections of the experimental spectra on the PCs a_{ij} (see Eq.(S1)) can be obtained by performing singular value decomposition (SVD) of the matrix formed by all the experimental spectra.[1, 3] Here for this purpose we used a combined dataset containing normalized Co K-edge XANES spectra for CoO_x, $Co_{2.25}Fe_{0.75}O_4$ and $CoFe_2O_4$, shown in **Figure 1a** in the main text and **Figure S1ab**. We do not include here the spectra for $Co_{0.25}Fe_{2.75}O_4$ due to the lower signal-to-noise ratio for this dilute sample. Nonetheless, once the PCs were obtained (based on the data for CoO_x, $Co_{2.25}Fe_{0.75}O_4$ and $CoFe_2O_4$), we found that all the spectra for $Co_{0.25}Fe_{2.75}O_4$ can also be accurately expressed as linear combinations of these PCs (**Figure S2**), suggesting that this sample does not contain spectroscopically unique species that are not already present in the datasets for CoO_x, $Co_{2.25}Fe_{0.75}O_4$ or $CoFe_2O_4$.

We note that if the spectra for the $Co_{0.25}Fe_{2.75}O_4$ sample are included in the construction of PCs, the current third principal component PC-3 becomes the fourth most significant PC (PC-4'), while the new third PC (PC-3') has no pronounced spectroscopic features (**Figure S6a**). However, the weight of PC-3' changes unsystematically from one sample and spectrum to another (**Figure S6b-e**). We thus conclude that the PC-3' corresponds to experimental noise, which in this particular case happens to provide a

larger contribution to the experimental data than the contribution of the physically meaningful PC-4'.

Supplementary Note 2: Details of neural network training and validation

In a heterogeneous material containing several different phases, and/or metal sites within the same phase, but with crystallographically non-equivalent environments, the total EXAFS spectrum $\chi(k)$ can be expressed as a sum of all contributions from all non-equivalent sites $\chi(k) = \sum_s w_s \chi_s(k)$. Here, w_s is the concentration of the s-th species (site), and $\chi_s(k)$ is the partial spectrum associated with the s-th species. Each $\chi_s(k)$ can, in turn, be expressed as a sum of contributions from different photoelectron scattering paths $\chi_s(k) = \sum_p \chi_{sp}(k)$. The summation includes both, single scattering as well as multiple scattering paths. Contributions of single scattering paths, in turn, can be directly linked to the radial distribution functions (RDFs) $g_{sp}(R)$ as

$$\chi_{sp}(k) = S_0^2 \sum_p \int_0^{+\infty} A_{sp}(k) g_{sp}(R) \sin\left(2kR + \varphi_{sp}(k)\right) \frac{dR}{R^2} \quad \text{(S2)}$$

Here A_{sp} and φ_{sp} represent the real and imaginary parts of the photoelectron scattering function, R is the interatomic distance between the absorbing metal atom and neighbouring atom of p-th type (e.g., oxygen or another metal), while S_0^2 is the amplitude reduction term accounting for the many-electronic effects. Considering that $g_{sp}(R)$ are not arbitrary functions, but must describe physically reasonable bond length distributions around the s-th species, the equation above defines the one-to-one correspondence between the EXAFS spectrum $\chi(k)$, and the set of all partial RDFs: $\{w_s g_{sp}(R)\} \leftrightarrow \chi(k)$. Importantly, the one-to-one correspondence holds only if $g_{sp}(R)$ for some species s cannot be expressed just by scaling the $g_{s'p}(R)$ function for some other species s' with some constant multiplier. Such scenario would result in an ambiguous situation, where an infinite number of $g_{s'p}(R)$ and $g_{sp}(R)$ combinations with different weights w_s and $w_{s'}$ would give the same total $\chi(k)$ spectrum. In our case, this condition means that the mapping between partial RDFs and EXAFS spectrum is unambiguous only if the analysis is extended to distant coordination shells: the bond length distributions within the first coordination shell are often very similar for different species s, corresponding, e.g., to different oxide phases.

The one-to-one correspondence between EXAFS spectra and the set of RDFs can be inverted by a neural network (NN) approach. Here the relationship between the spectral features and RDFs is established during NN training procedure, where a large set of theoretical EXAFS spectra with known corresponding RDFs is used to optimize NN parameters.

To construct a dataset for NN training, we follow the approach introduced in our previous works.[3-5] We first generate pairs of theoretical site-specific EXAFS spectra and corresponding RDFs for all unique metal sites in a set of relevant Co and Fe oxides, hydroxides and oxyhydroxides. Here we consider octahedrally-coordinated Co species in rocksalt-type Co(II) oxide (CoO-*rs*), $CoOH_2$ and CoOOH, tetrahedrally coordinated Co species in wurtzite type CoO (CoO-*w*), as well as tetrahedrally and octahedrally

coordinated Co sites in Co_3O_4, octahedrally-coordinated Fe species in FeO, α-Fe_2O_3, FeOOH, and tetrahedrally- and octahedrally-coordinated Fe sites in spinel-type Fe_3O_4 and γ-Fe_2O_3. For each of these well-defined reference compounds we sampled atomic configurations using molecular dynamics (MD) or Monte Carlo (MC) methods with empirical force field models. Whenever available, we used force field models from the literature, including Buckingham-type potential developed by Lewis and Catlow, used here for rocksalt-type FeO and CoO,[6] potential developed by Cooke et al that we used for γ-Fe_2O_3,[7] potential for α-Fe_2O_3 from Erlebach at al,[8] and an exponential shell potential for Co_3O_4 from Hu et al.[9] For the structure models, for which the potentials were not available in the literature, we have found that good results can be obtained by using simple Lennard-Jones type potentials $V_{LJ} = 4\varepsilon\left(\left(\frac{\sigma}{r}\right)^{12} - \left(\frac{\sigma}{r}\right)^{6}\right)$, where the σ parameter is chosen to match the equilibrium distance between the nearest neighbors for given reference material, and ε parameter (potential depth) is optimized to ensure that the EXAFS spectra, calculated with this potential, match as well as possible the available room temperature EXAFS spectra for this reference material. Clearly, this simple approach cannot yield force field model that is able to reproduce all physical properties of the material. Nonetheless, it is sufficient for our purposes, since it allows us to generate realistic-looking EXAFS spectra and corresponding RDFs that are needed for NN training. MD and MC simulations were carried out using *Gulp* code.[10]

MD and MC simulations have been carried out at different temperatures, to account for different degrees of structural disorder. The obtained atomic coordinates in the MD and MC models were further isotropically rescaled in the range between 0.95% and 1.05%, to account for possible variations in the lattice constants. For each of the resulting ca. 20000 structure models we calculated the corresponding partial metal-oxygen and metal-metal RDFs.

Theoretical EXAFS spectra for structures sampled by MD and MC methods, were calculated using *EvAX* code,[11] which itself uses *FEFF-8* code[12] for *ab initio* calculations of EXAFS spectra. Pairs of the calculated site-specific EXAFS spectra $\chi_i(k)$ and partial RDFs sets $\vec{G}_i$ were then constructed, where $\vec{G}_i$ is a vector consisting of four concatenated partial RDFs, corresponding to the M_{Th}-O, M_{Th}-M, M_{Oh}-O and M_{Oh}-M bond length distributions. Here M is either Fe or Co, and M_{Th} and M_{Oh} denote tetrahedrally or octahedrally coordinated metal site, respectively. Each individual RDF is parametrized as a histogram of bond lengths in the range between R_{min} = 1.0 Å and R_{max} = 4.0 Å, with a histogram bin width of 0.06 Å. At this point, clearly, at least half of the elements in each $\vec{G}_i$ vector are 0, because for the tetrahedrally coordinated central atoms M_{Oh}-O and M_{Oh}-M RDFs are equal to 0, while for the octahedrally coordinated central atoms M_{Th}-O and M_{Th}-M RDFs are equal to 0.

The generated site-specific spectra and corresponding site-specific RDFs corresponding to unique sites in pure compounds were mixed linearly, to obtain 10000 pairs of spectra and RDFs for mixtures. For this purpose we randomly selected three $\{\chi_i(k), \vec{G}_i\}$, and constructed their linear combination with random weights w_{ji}, giving us spectrum for a

mixture of species $\tilde{\chi}_j(k) = \sum_i w_{ji}\chi_i(k)$ and the corresponding concatenated RDF vector $\tilde{G}_j(k) = \sum_i w_{ji}\vec{G}_i$. We repeated this process 10000 times.

One should note here that our approach will be able to interpret EXAFS spectra correctly in the case when they correspond to the structures that are not too different from those considered when constructing the training data set. In the present case these are, for instance, mixtures of structural motifs encountered in common Co and Fe oxide materials. Some hypothetical amorphous structures with unique bonding motifs can thus be missed. Unfortunately this limitation is unavoidable. In fact, the extraction of RDFs from EXAFS spectra is, in general, an ill-defined problem, if no additional assumptions are made about which solutions might be feasible. This is because many different RDFs can produce similar EXAFS spectra if no restrictions on the shapes of the RDFs are imposed. As a result, one needs to define, which solutions to consider. In our approach this is done automatically based on the chosen training data set, limiting thus the applicability of the method to a certain class of materials.

NN is then constructed as a mathematical function $H(\vec{\theta}, \chi) \to G$ that depends on a large set of parameters $\vec{\theta}$ and maps the EXAFS spectrum χ, provided as its input, to some output vector G. During the NN training, we provide the spectra $\tilde{\chi}_j(k)$ from the training set as inputs for the function *H,* and tune the weights $\vec{\theta}$, so that the NN outputs match as well as possible the known corresponding $\tilde{G}_j$ vectors. After the training is completed, the weights $\vec{\theta}$ are fixed, and the NN can take as input experimental EXAFS spectra, providing as outputs the corresponding sets of partial RDFs.

NN for EXAFS data interpretation was constructed and trained similarly as in our previous works.[4, 5] Our neural network consists of an input layer, several hidden layers, and the output layer. The nodes in the input layer are initialized by the analyzed EXAFS data. The output layer yields the vector describing concatenated partial RDFs. The nodes in the hidden layers increase the flexibility of the mathematical model, represented by NN, and allow it to map the strongly non-linear relationships between input and output vectors.

In our previous works, before being provided to NN as an input, an EXAFS spectrum was pre-processed using Morlet wavelet transform.[4, 13] In this work, we used instead the short time Fourier transform (STFT), which can be considered as a special case of Morlet wavelet transform, where the width of wavelet function is fixed and not scaled with frequency. The STFT was carried out in a *k*-range between 3 and 9 $Å^{-1}$. We have checked the effect of the used *k*-range on the stability and accuracy of NN predictions, and have found no significant improvements, if the *k*-range was extended to 12 $Å^{-1}$. For the NN input, we used selected STFT coefficients (both real and imaginary parts) that exhibited the largest sensitivity to the details of local structure. In total, the NN input layer contained 2165 nodes.

The output layer of our NN represents four concatenated RDFs (bond length histograms), corresponding to the M_{Th}-O, M_{Th}-M, M_{Oh}-O and M_{Oh}-M bond length distributions. The output layer of our NN contains 4 x 51 = 204 nodes.

In addition to input and output layers, our NN contained also three hidden layers with 2000 nodes in each. Number of layers and nodes, as well as other hyperparameters of

our NN were optimized in a manual grid search. The NN construction and training was performed using *Wolfram Mathematica*[14] version 13.0. See Ref.[4] for the technical details of NN training.

To estimate the uncertainties, we constructed and trained independently 10 different NNs. The NN-EXAFS results reported here and in the main text are average predictions of these 10 NNs, while the standard deviations of NN predictions are used as estimators of statistical uncertainty.

To validate the NNs, and to assess the systematic errors, we test the NN-EXAFS method on a set of reference spectra for standard materials, where the RDFs can be obtained independently by using reverse Monte Carlo (RMC) simulations.[11, 15] RMC is an EXAFS fitting approach, where the atomic coordinates within 3D structure model of the material are iteratively optimized, until a good agreement is achieved between experimental EXAFS spectrum, and corresponding theoretical EXAFS spectrum, calculated for the structure model. As shown in **Figure S12**, RMC allows one to obtain structure models in an excellent agreement with experimental EXAFS data, accounting also for the contributions of multiple scattering effects, distant coordination shells and non-Gaussian shapes of bond length distributions. As demonstrated in **Figure S10** and **Figure 4** in the main text, the RDFs, yielded by NN-EXAFS method, agree well with the RDFs from RMC-EXAFS simulations.

Supplementary Note 3: Effect of the Fe K-edge EXAFS on the Co K-edge EXAFS

When discussing EXAFS spectra in materials featuring elements that are neighbors in the Periodic Table, especially if the concentration of the lighter element (Fe in our case) is much higher that that of the heavier element (Co in our case), a ”leaking” of EXAFS oscillations of the lighter element into the EXAFS spectrum of the heavier element can sometimes be observed.[16] This could result in the distortions of the Co K-edge EXAFS spectra for Co-poor materials.

To check the importance of this effect in our samples, and to test, whether the Fe K-edge EXAFS oscillations affect the shape of Co K-edge EXAFS, we constructed an artificial X-ray absorption spectrum, where we added the normalized reference spectra for γ-Fe_2O_3 and Co_3O_4, collected at Fe K-edge and Co K-edge, respectively. As discussed in the main text, these reference materials represent well the local structure in our catalysts. The weights of Fe and Co spectra in these synthetic spectra were varied to model the effect of different Fe to Co ratios in our samples. From these synthetic spectra we then extracted Co K-edge EXAFS, and compared it with the true Co K-edge EXAFS spectrum for Co_3O_4 (**Figure S16**). As one can see, even for the highest Fe to Co ratio (11:1, corresponding to the Fe to Co ratio in our $Co_{0.25}Fe_{2.75}O_4$ sample), the leaking of Fe K-edge EXAFS into Co K-edge EXAFS results only in very minor distortions of Co K-edge EXAFS features. This effect also certainly cannot affect our main conclusion for the $Co_{0.25}Fe_{2.75}O_4$ sample, namely. For samples with lower Fe to Co ratios, the leaking of Fe K-edge EXAFS into Co K-edge EXAFS is completely negligible.

Supplementary Figures

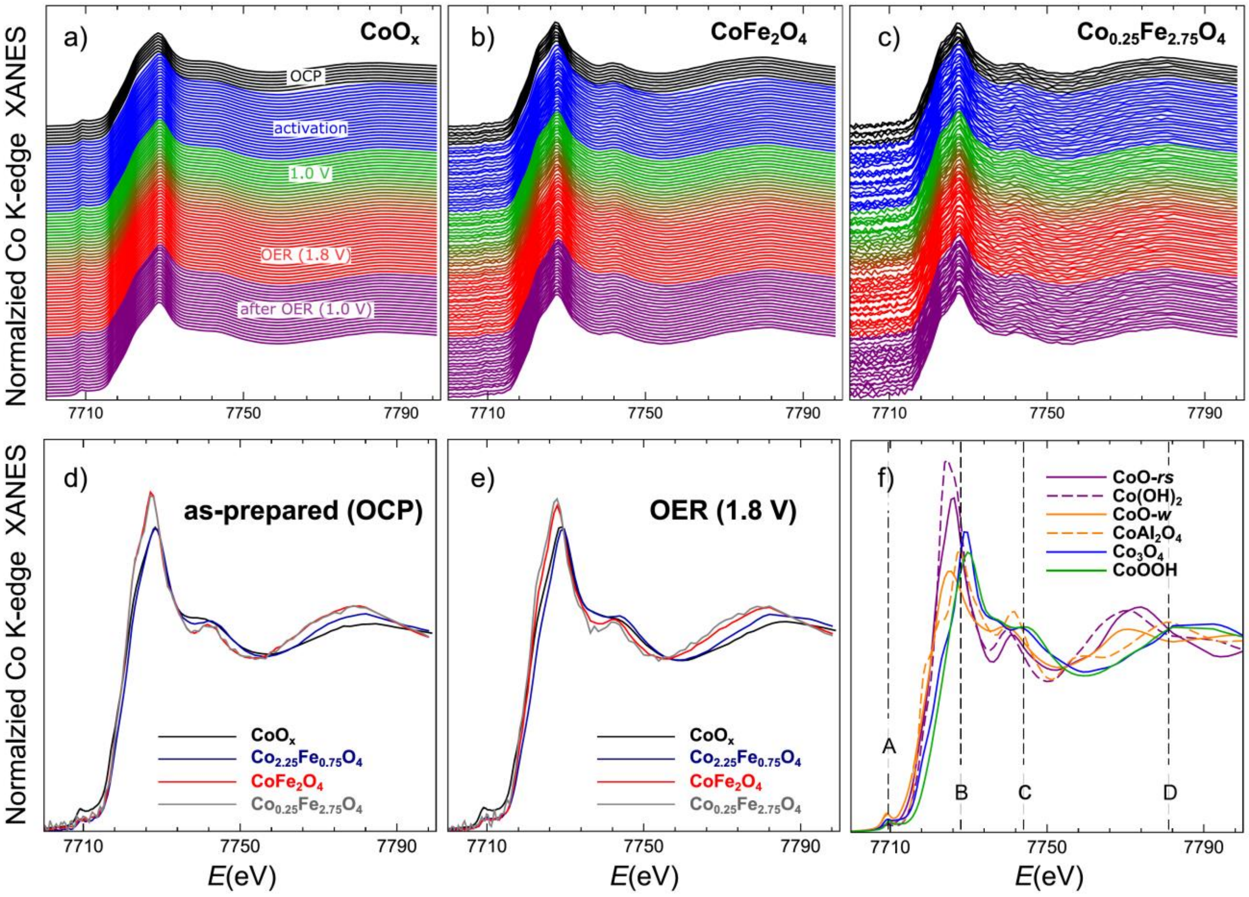

Figure S1. Evolution of Co K-edge XANES spectra during activation and under OER conditions for CoOx (a), CoFe2O4 (b) and Co0.25Fe2.75O4 (c) catalysts. Spectra are shifted vertically for clarity. Comparison of Co K-edge XANES spectra for as-prepared CoO_x, $Co_{2.25}Fe_{0.75}O_4$, $CoFe_2O_4$ and $Co_{0.25}Fe_{2.75}O_4$ catalysts under open circuit potential (d) and for catalysts under OER conditions at $1.8V_{RHE}$(e). Reference spectra for rocksalt-type CoO, $Co(OH)_2$, wurtzite-type CoO, spinel type $CoAl_2O_4$ and Co_3O_4 and for CoOOH (f).

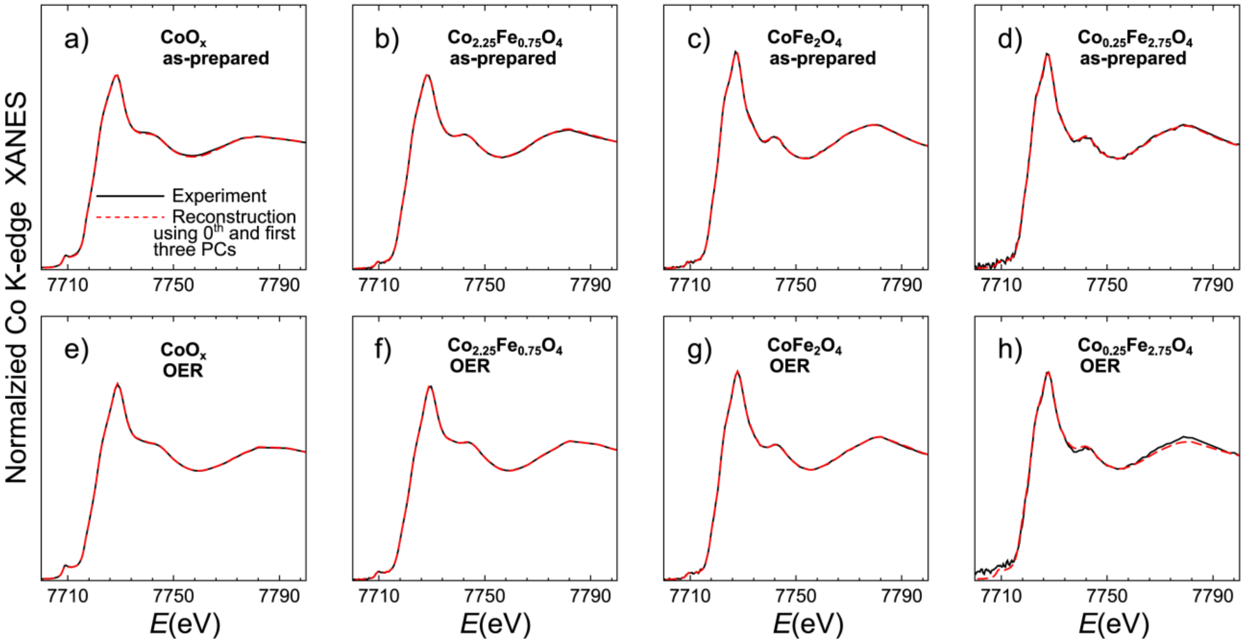

Figure S2. Experimental Co K-edge XANES spectra and the results of spectra reconstruction using the average spectrum and the first three principal components for as-prepared CoO_x, $Co_{2.25}Fe_{0.75}O_4$, $CoFe_2O_4$ and $Co_{0.25}Fe_{2.75}O_4$ catalysts under open circuit potential (a-d) and for catalysts under OER conditions at $1.8V_{RHE}$(e-h).

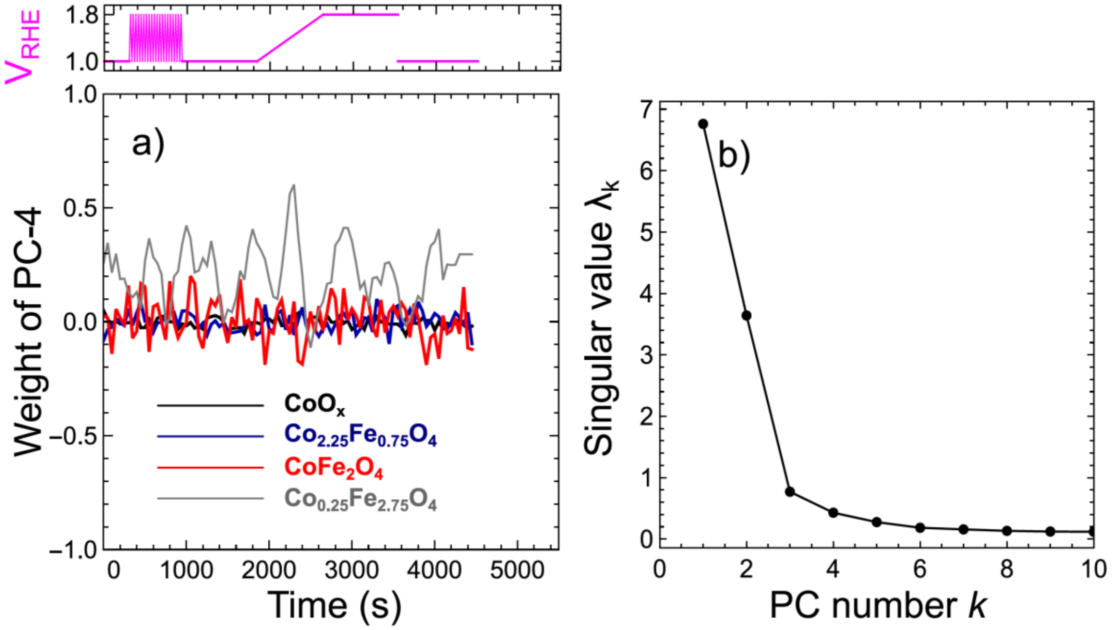

Figure S3. (a) Evolution of the weight of the 4th principal component during the activation and under OER conditions for $Co_xFe_yO_z$ catalysts. (b) Scree plot characterizing the relative importance of PCs by their corresponding singular values λ_k.

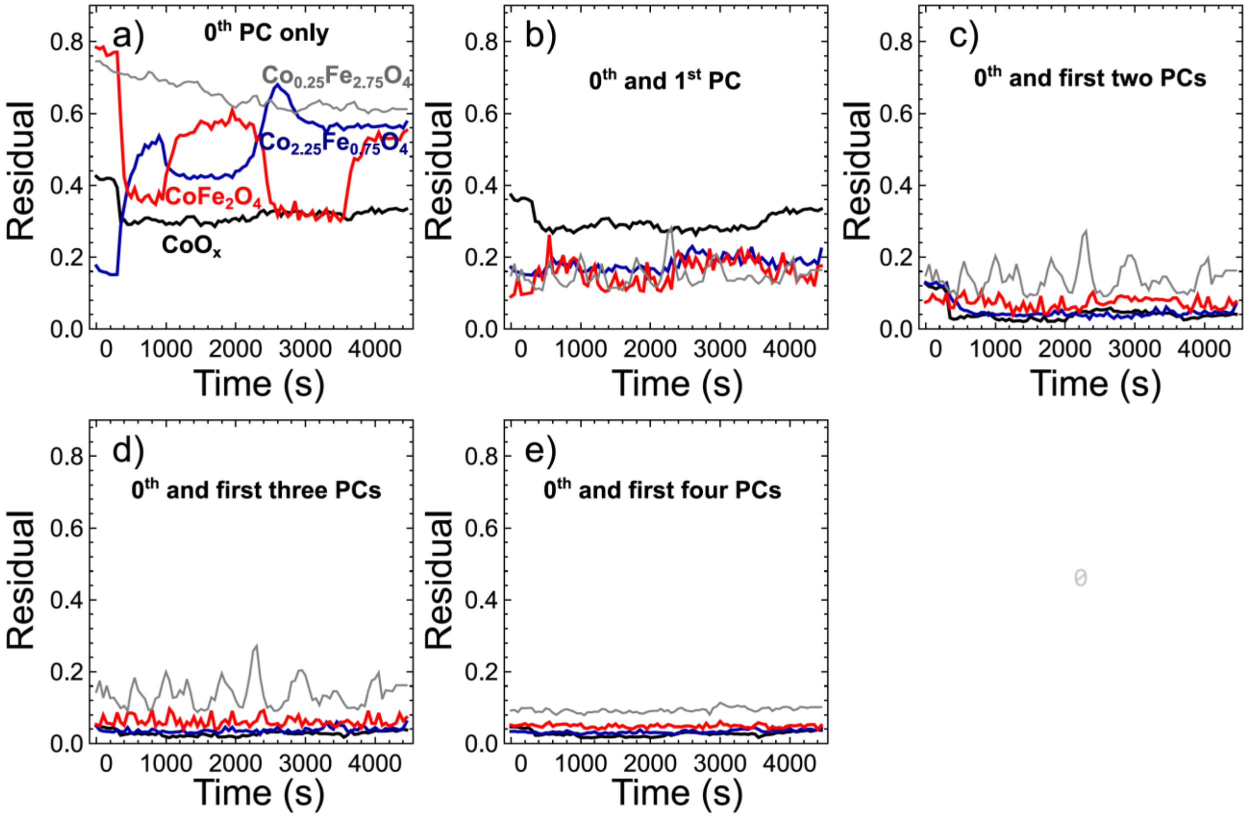

Figure S4. Residual of the principal component decomposition for Co K-edge XANES spectra collected during the activation and under OER conditions for $Co_xFe_yO_z$ catalysts, if in the decomposition one includes 0 (a), one (b), two (c), three (d) or four (e) principal components.

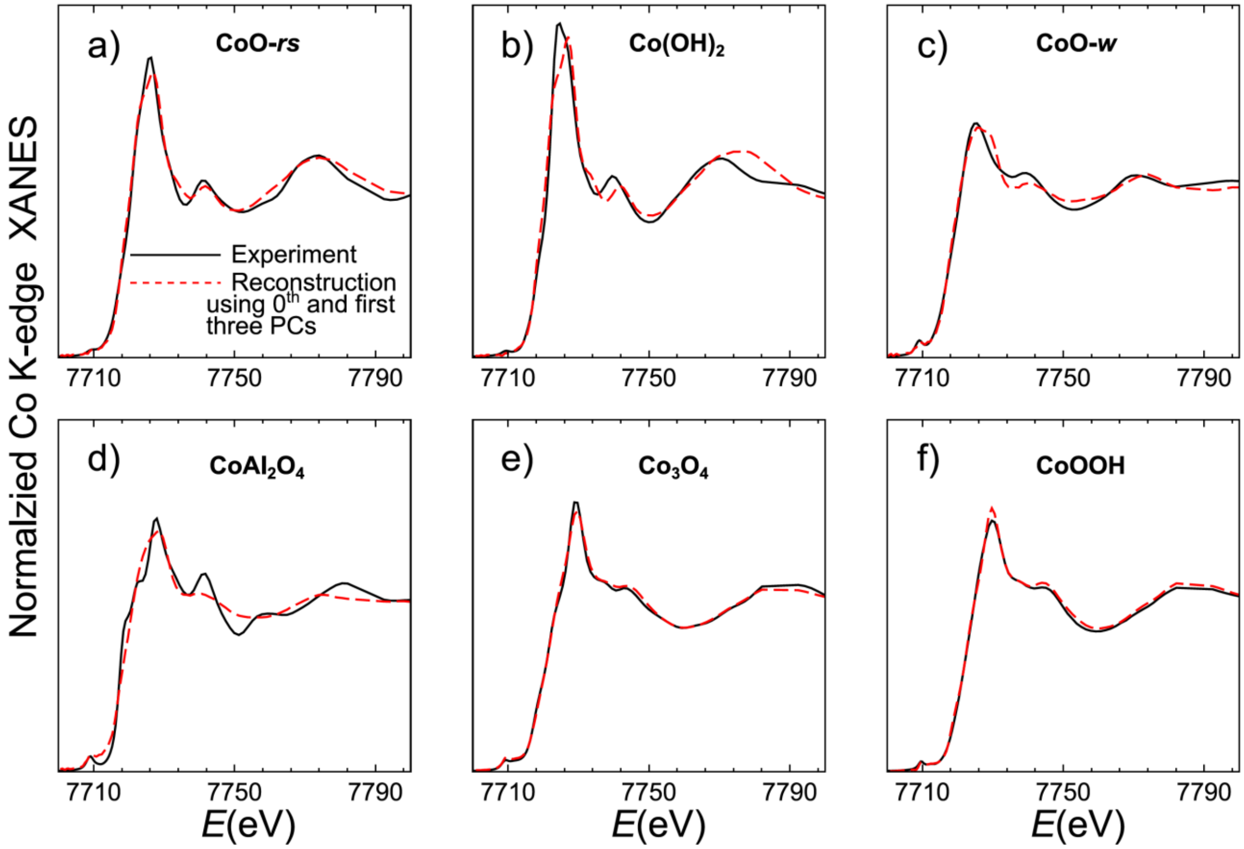

Figure S5. Experimental Co K-edge XANES spectra for standard reference materials and the results of spectra reconstruction using the average spectrum and the first three principal components, as extracted from *operando* data for $Co_xFe_yO_z$ catalysts. Results for rocksalt-type CoO (a), $Co(OH)_2$ (b), wurtzite-type CoO (c), spinel type $CoAl_2O_4$ (d) and Co_3O_4 (e) and for CoOOH (f) are shown.

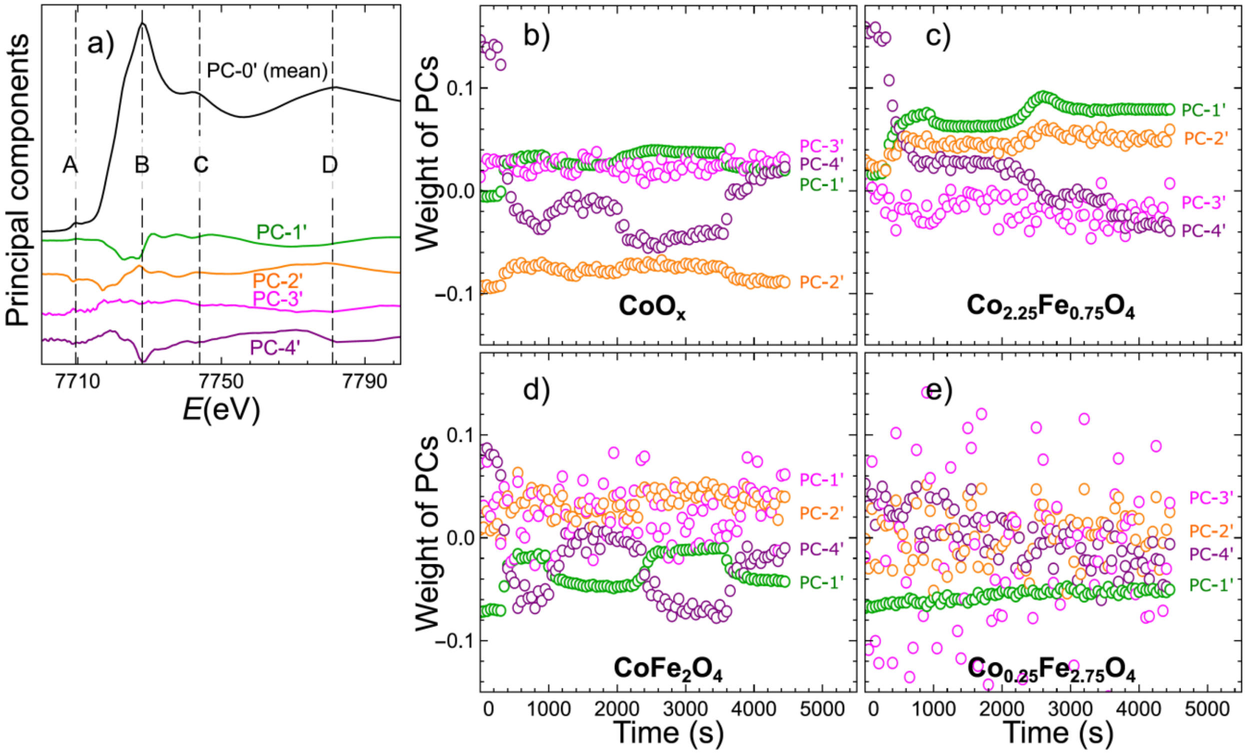

Figure S6. (a) Average spectrum and the first four principal components, as obtained from the combined Co K-edge XANES data set for $Co_xFe_{3-x}O_4$ catalysts collected under activation conditions as well as during and after OER at +1.8 V_{RHE}. In contrast to Figure 1a in the main text, spectra for $Co_{0.25}Fe_{2.75}O_4$ were used together with those for CoO_x, $Co_{2.25}Fe_{0.75}O_4$ to constrict the PCs. (b-e) Evolution of the weights of the first, second, third and fourth PCs shown in (a) during the activation and under OER conditions at +1.8 V_{RHE} for CoO_x (b), $Co_{2.25}Fe_{0.75}O_4$ (c), $CoFe_2O_4$ (d) and $Co_{0.25}Fe_{2.75}O_4$ catalysts (e).

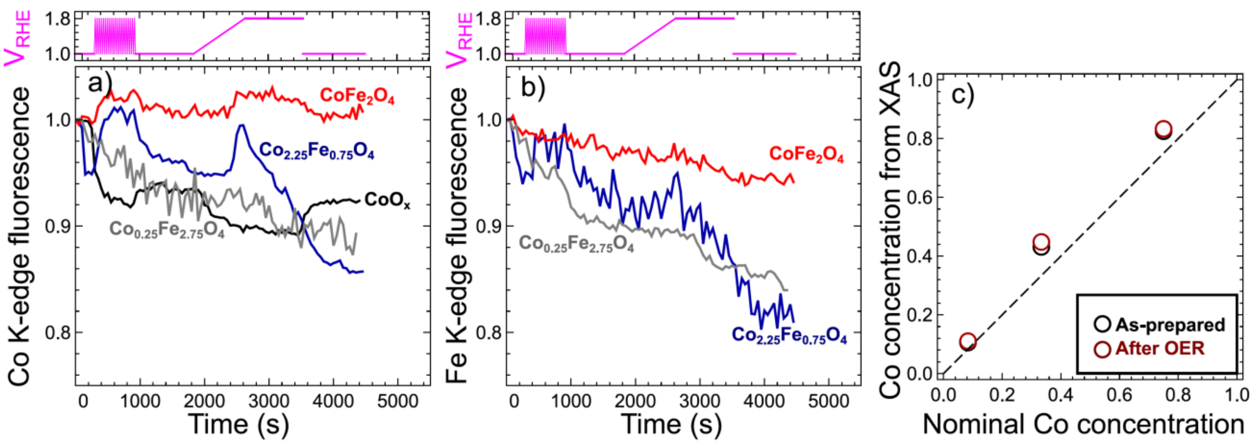

Figure S7. Relative fluorescence intensity jump at Co K-edge (a) and Fe K-edge (b) for CoO_x, $Co_{2.25}Fe_{0.75}O_4$, $CoFe_2O_4$ and $Co_{0.25}Fe_{2.75}O_4$ catalysts during the activation and under OER conditions. Concentration of Co, as calculated from the intensities of Co K-edge and Fe K-edge fluorescence intensity jumps for as-prepared samples and samples after OER, compared to the nominal Co concentration.

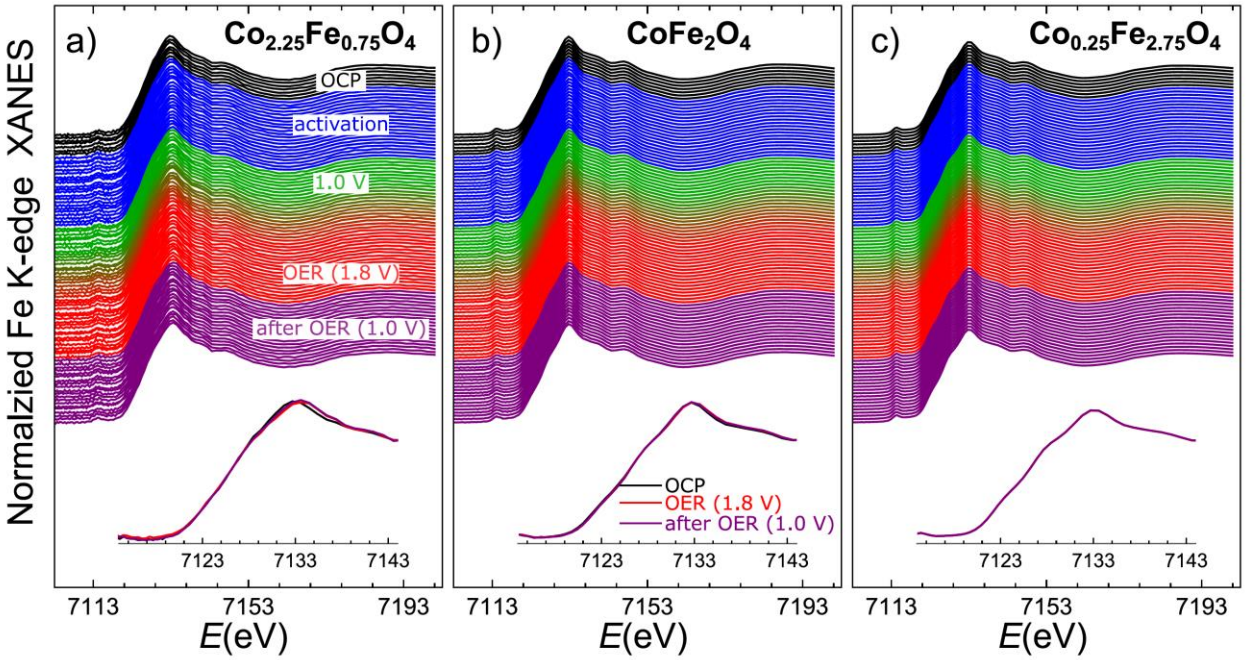

Figure S8. Evolution of Fe K-edge XANES spectra during activation and under OER conditions for $Co_{2.25}Fe_{0.75}O_4$ (a), $CoFe_2O_4$ (b) and $Co_{0.25}Fe_{2.75}O_4$ (c) catalysts. Spectra are shifted vertically for clarity. Insets show comparison of representative spectra for as-prepared samples, samples under OER conditions and for samples after OER. Each depicted spectrum was obtained after averaging the QXAFS data collected within 50 s.

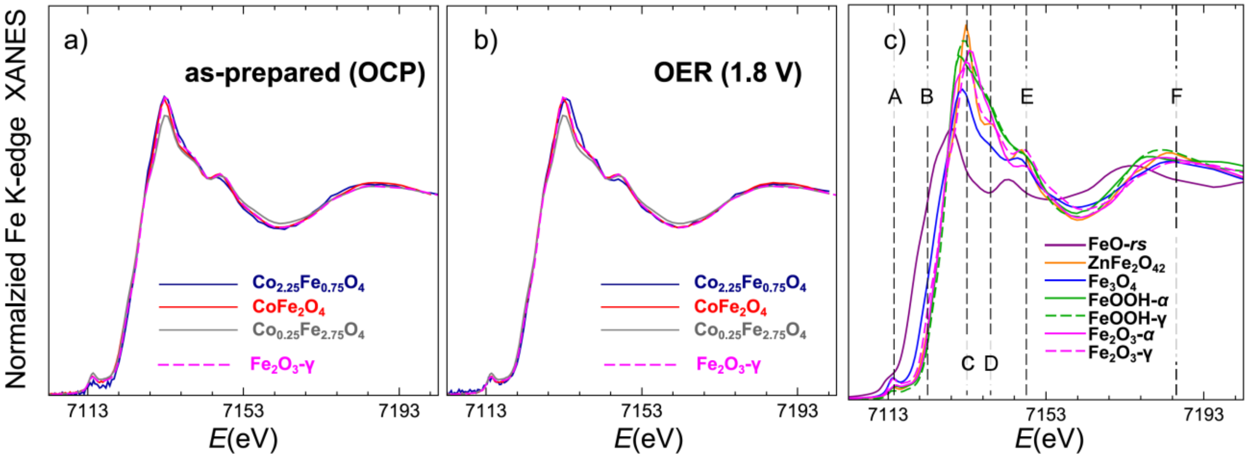

Figure S9. Comparison of Fe K-edge XANES spectra for as-prepared $Co_{2.25}Fe_{0.75}O_4$, $CoFe_2O_4$ and $Co_{0.25}Fe_{2.75}O_4$ catalysts under open circuit potential (a) and for catalysts under OER conditions at $1.8V_{RHE}$ (b). Reference spectra for rocksalt-type FeO, spinel type $ZnFe_2O_4$, Fe_3O_4 and Fe_2O_3-*γ*, as well as for FeOOH-*α*, FeOOH-*γ* and Fe_2O_3-*α* (c).

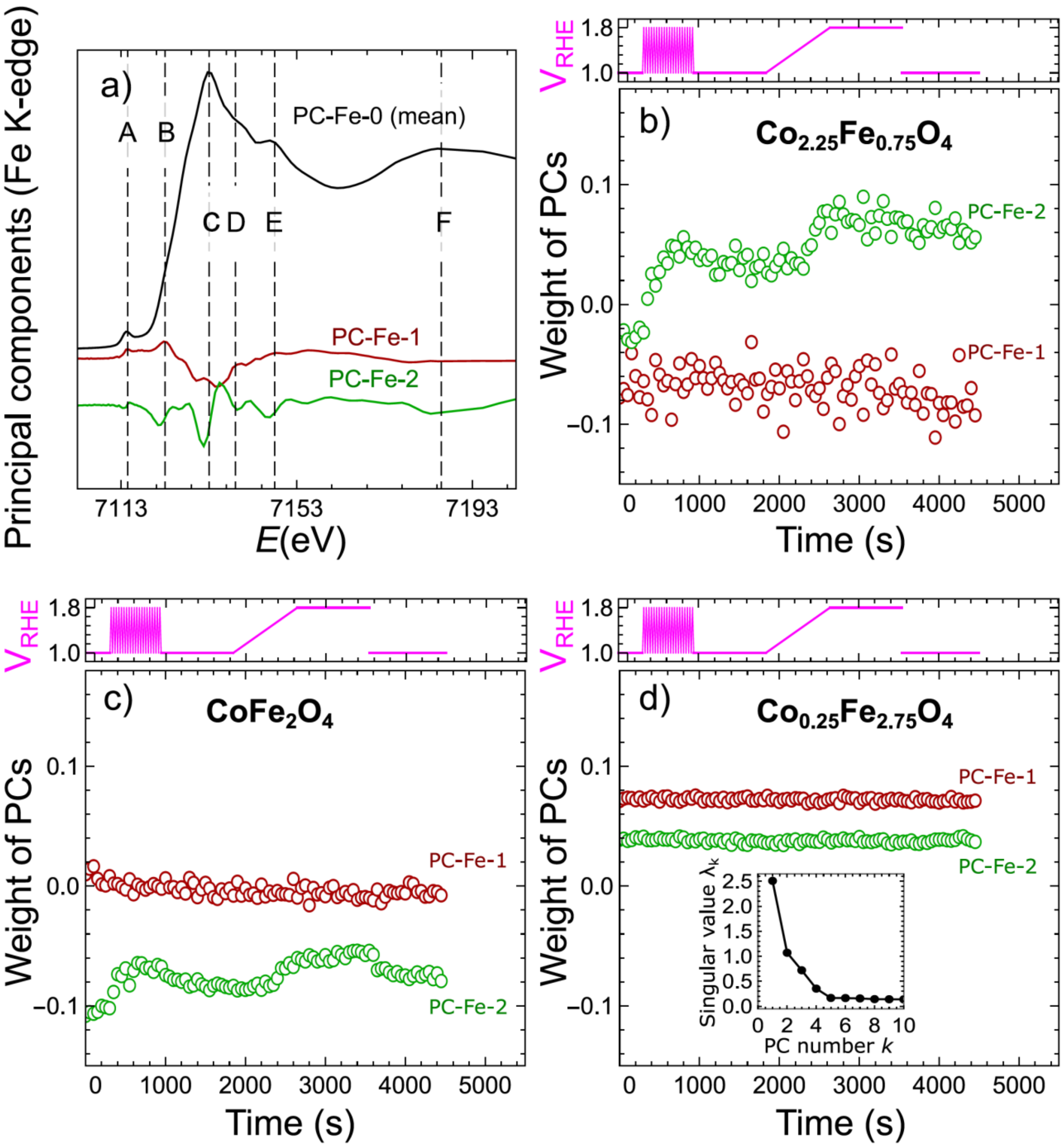

Figure S10. Principal component analysis for Fe K-edge XANES data. Average spectrum and the first two principal components, as obtained from the combined Fe K-edge XANES data set for $Co_xFe_yO_z$ catalysts (a). Evolution of the weights of the first and second principal component during the activation and under OER conditions for $Co_{2.25}Fe_{0.75}O_4$ (b), $CoFe_2O_4$ (c) and $Co_{0.25}Fe_{2.75}O_4$ catalysts (d). Inset in (d) shows the scree plot for the principal components determined from the Fe K-edge XANES data.

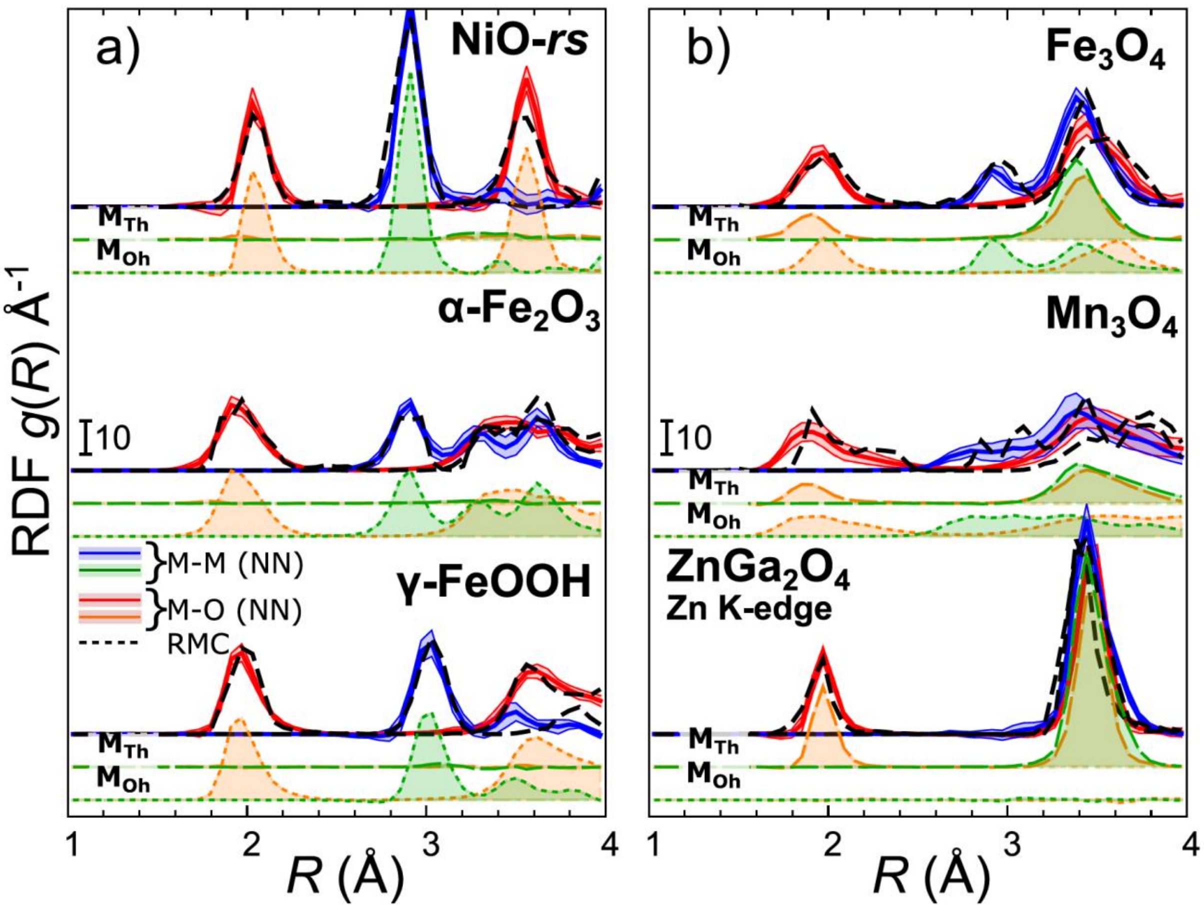

Figure S11. Validation of the NN-EXAFS method using RMC simulations and EXAFS spectra for reference materials (NiO, α-Fe_2O_3 and γ-FeOOH (a), and spinel-type Fe_3O_4, Mn_3O_4 and $ZnGa_2O_4$ (b). The total M-O and M-M RDFs, calculated from the same experimental Fe, Mn, Zn or Ni K-edge EXAFS using RMC and NN-EXAFS methods are shown, as well as partial M_{Th}-O, M_{Th}-M, M_{Oh}-O and M_{Oh}-M RDFS, extracted by NN-EXAFS method. RDFs are shifted vertically for clarity. Blue lines correspond to the total metal-metal radial RDFs. Shaded blue areas indicate the uncertainty of the NN results. Green lines indicate the partial RDFs calculated for tetrahedrally coordinated and octahedrally coordinated metal sites. Red lines correspond to the total metal-oxygen RDFs, with the NN uncertainty indicated as red shaded areas. The orange lines correspond to the partial RDFs calculated for tetrahedrally coordinated and octahedrally coordinated metal sites

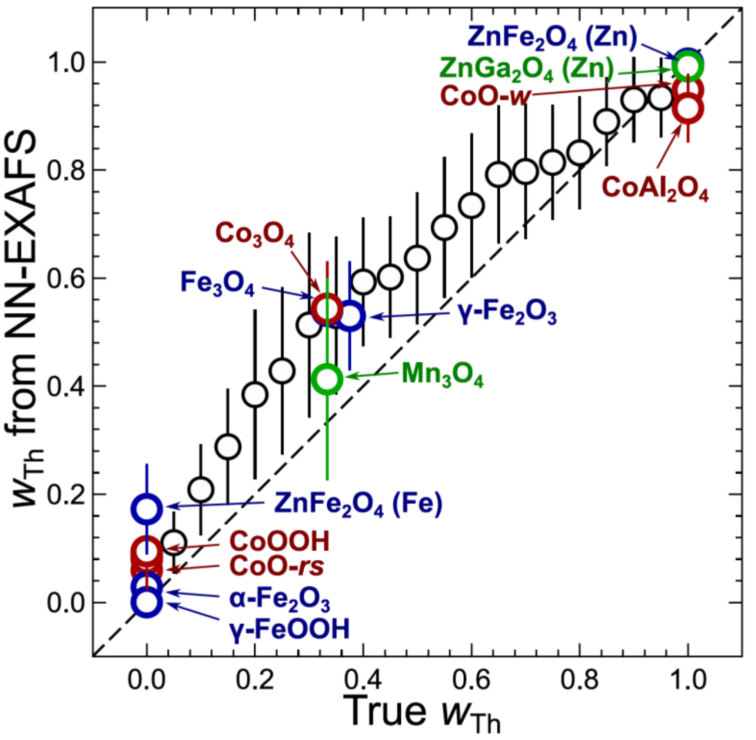

Figure S12. Validation of the NN-EXAFS method using EXAFS spectra for reference materials. The fractions of tetrahedrally coordinated metal sites, as obtained from the NN-EXAFS analysis of Fe K-edge (blue circles), Co K-edge (red circles) and Mn K-edge and Zn K-edge (green circles) reference materials. Black circles show results obtained for synthetic spectra, constructed as linear combinations of Co K-edge EXAFS spectra for wurtzite-type CoO (tetrahedrally coordinated Co), and CoOOH (octahedrally coordinated Co).

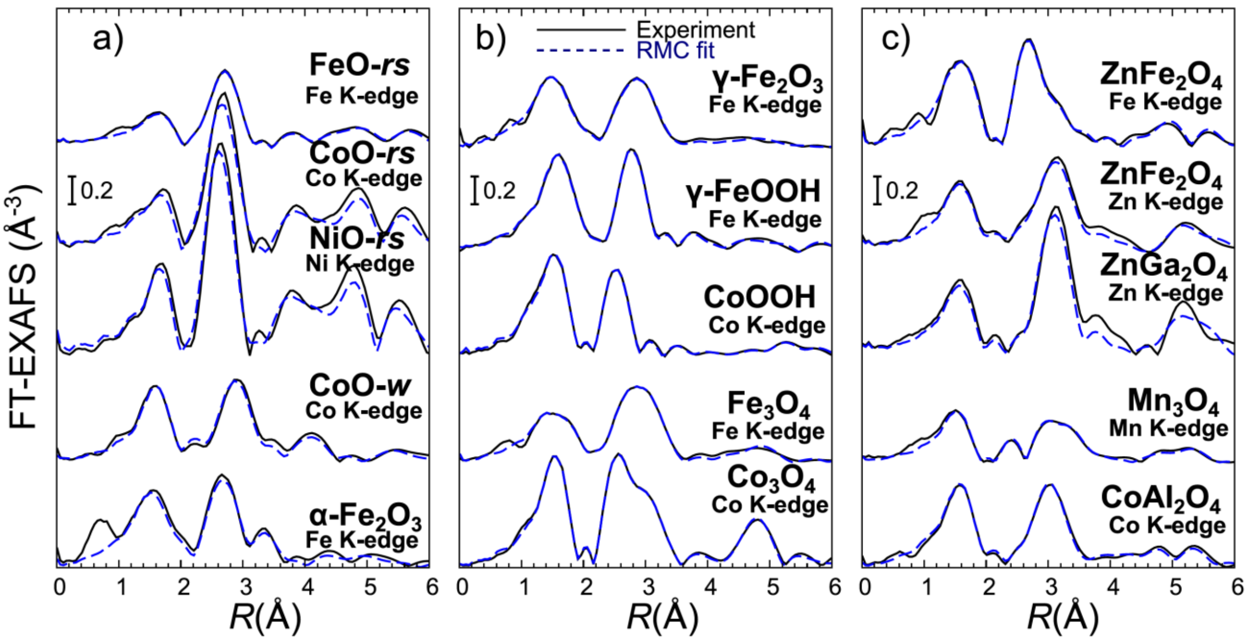

Figure S13. Reverse Monte Carlo (RMC) fits of EXAFS spectra for reference materials: comparison of Fourier-transformed experimental EXAFS data with the theoretical EXAFS spectra calculated for the final structure models obtained in RMC simulations. Spectra are shifted vertically for clarity.

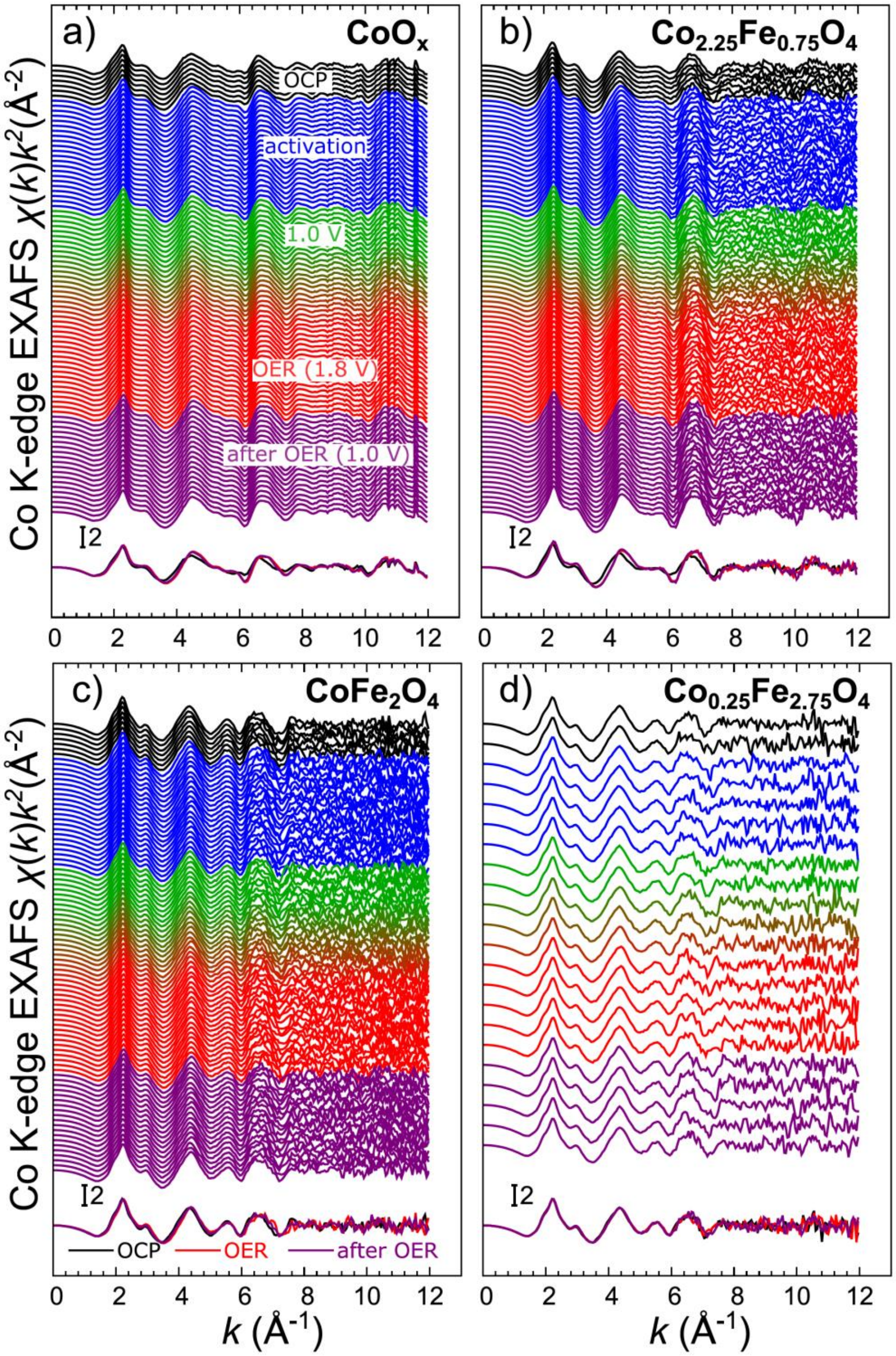

Figure S14. Evolution of Co K-edge EXAFS spectra during the activation and under OER conditions at $1.8V_{RHE}$ for CoO_x (a), $Co_{2.25}Fe_{0.75}O_4$ (b), $CoFe_2O_4$ (c) and $Co_{0.25}Fe_{2.75}O_4$ catalysts (d). Spectra are shifted vertically for clarity. Insets compare representative spectra for as-prepared samples, samples under OER conditions and for samples after OER. Each depicted spectrum is obtained after averaging the QXAFS data collected within 50 s (for panels (a,b, c)), and within 200 s for spectra in panel (d).

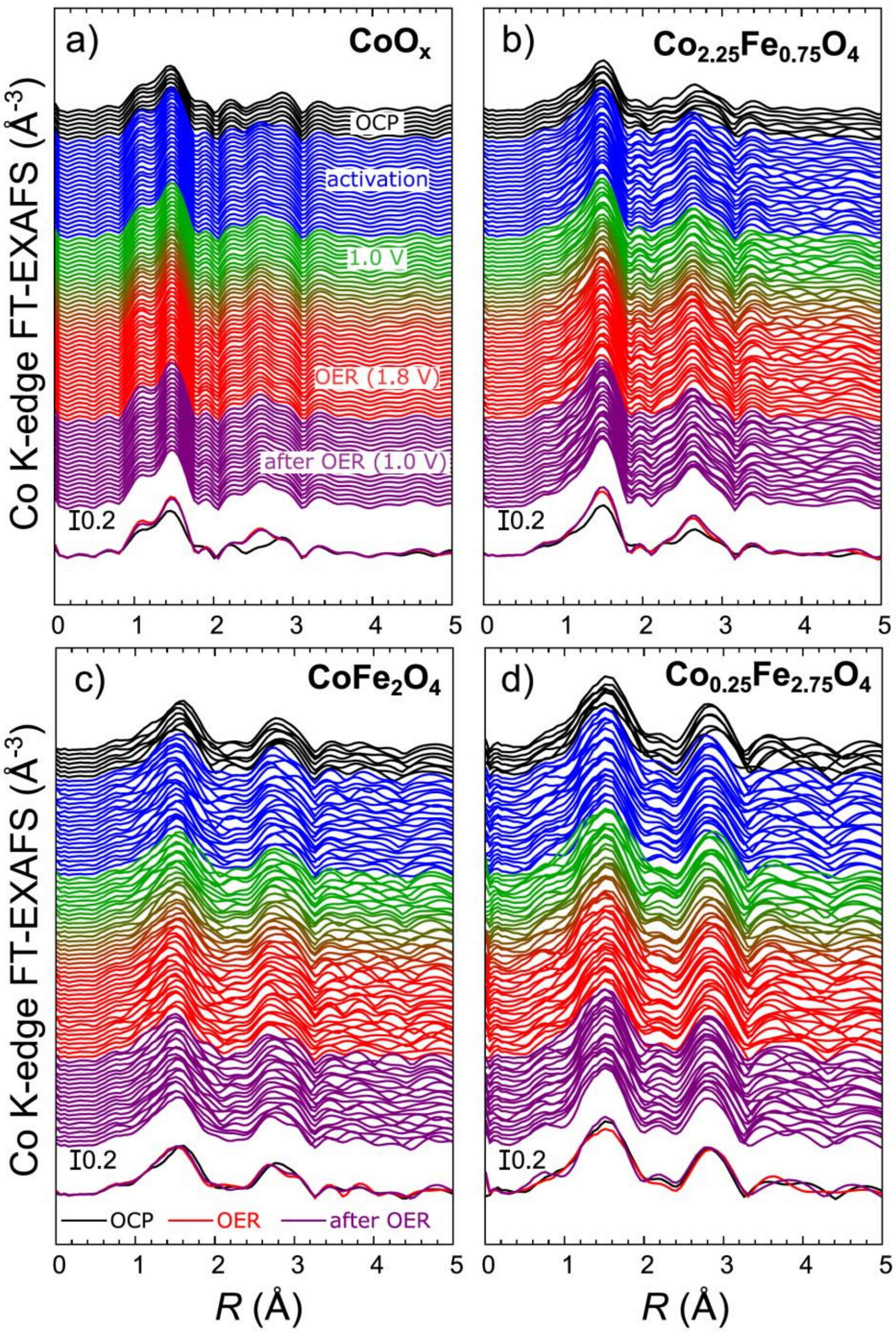

Figure S15. Evolution of Fourier transformed (FT) Co K-edge EXAFS spectra during activation and under OER conditions at $1.8V_{RHE}$ for CoO_x (a), $Co_{2.25}Fe_{0.75}O_4$ (b), $CoFe_2O_4$ (c) and $Co_{0.25}Fe_{2.75}O_4$ catalysts (d). Spectra are shifted vertically for clarity. Insets compare representative spectra for as-prepared samples, samples under OER conditions and for samples after OER. Fourier transform was carried out in the k-range between 1 and 12 $Å^{-1}$ in the panels (a,b, c), while for Co-poor sample in panel (d) shorter k-range was used (between 1 and 9 $Å^{-1}$) due to the lower signal-to-noise ratio. Each depicted spectrum is obtained after averaging the QXAFS data collected within 50 s (for panels (a,b, c)), and within 200 s for spectra in panel (d).

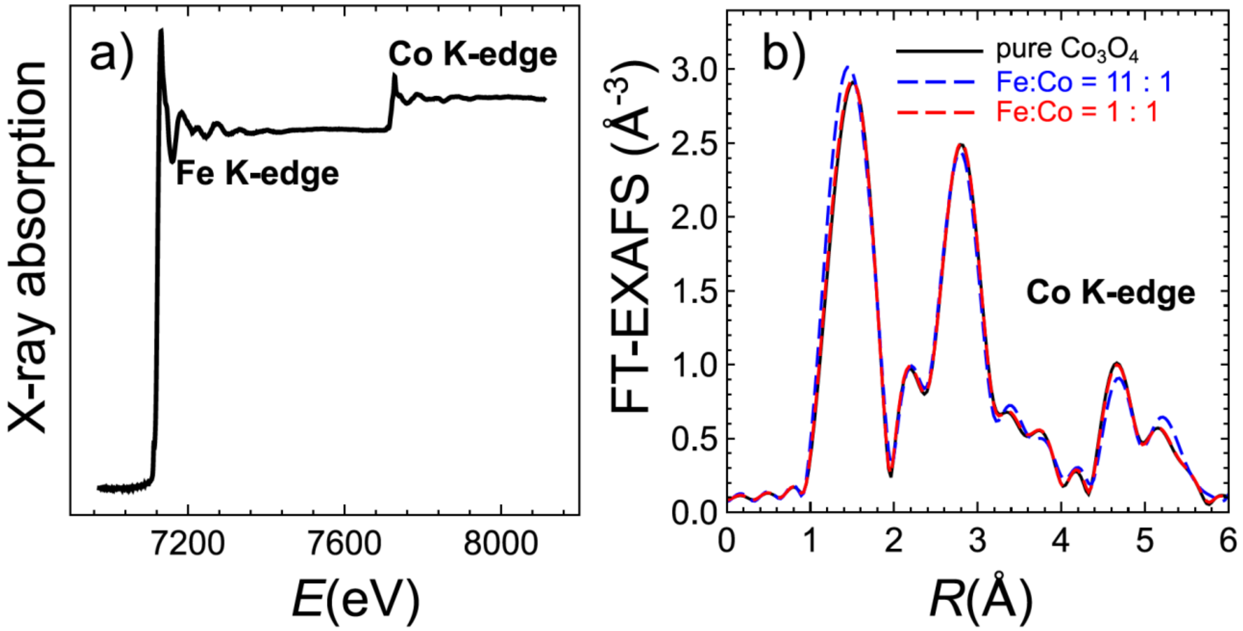

Figure S16. (a) Synthetic XAS spectrum, constructed by adding normalized reference spectra of γ-Fe_2O_3 and Co_3O_4, collected at Fe K-edge and Co K-edge respectively. The weights of Fe K-edge and Co K-edge spectrum are 11 to 1, mimicking the Fe to Co ratio in $Co_{0.25}Fe_{2.75}O_4$ sample. (b) Comparison of the Fourier transformed Co K-edge EXAFS spectra for Co_3O_4 with that for the artificial spectrum shown in (a), as well as for the artificial XAS spectrum constructed by adding spectra of γ-Fe_2O_3 and Co_3O_4 with the ratio 1 to 1.

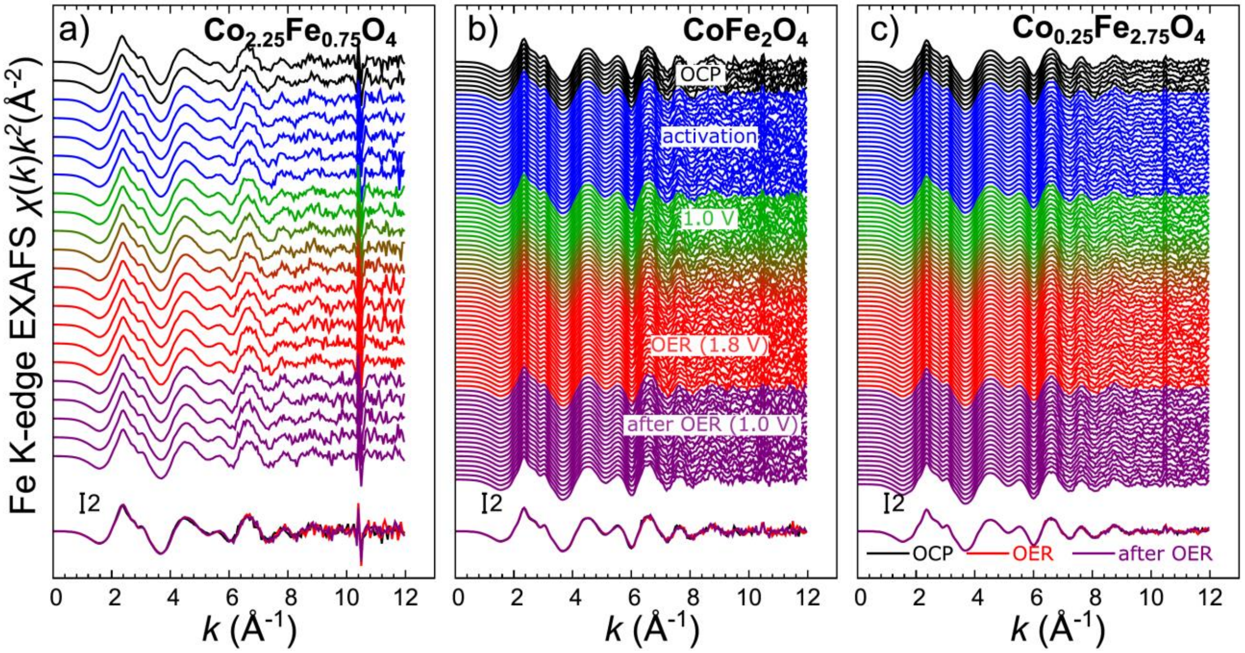

Figure S17. Evolution of Fe K-edge EXAFS spectra during the activation and under OER conditions at 1.8V$_{RHE}$ for $Co_{2.25}Fe_{0.75}O_4$ (a), $CoFe_2O_4$ (b) and $Co_{0.25}Fe_{2.75}O_4$ (c) catalysts. Spectra are shifted vertically for clarity. Insets compare representative spectra for the as-prepared samples, samples under OER conditions and for samples after OER. Each depicted spectrum is obtained after averaging the QXAFS data collected within 50 s (for panels (b, c)), and within 200 s for spectra in panel (a).

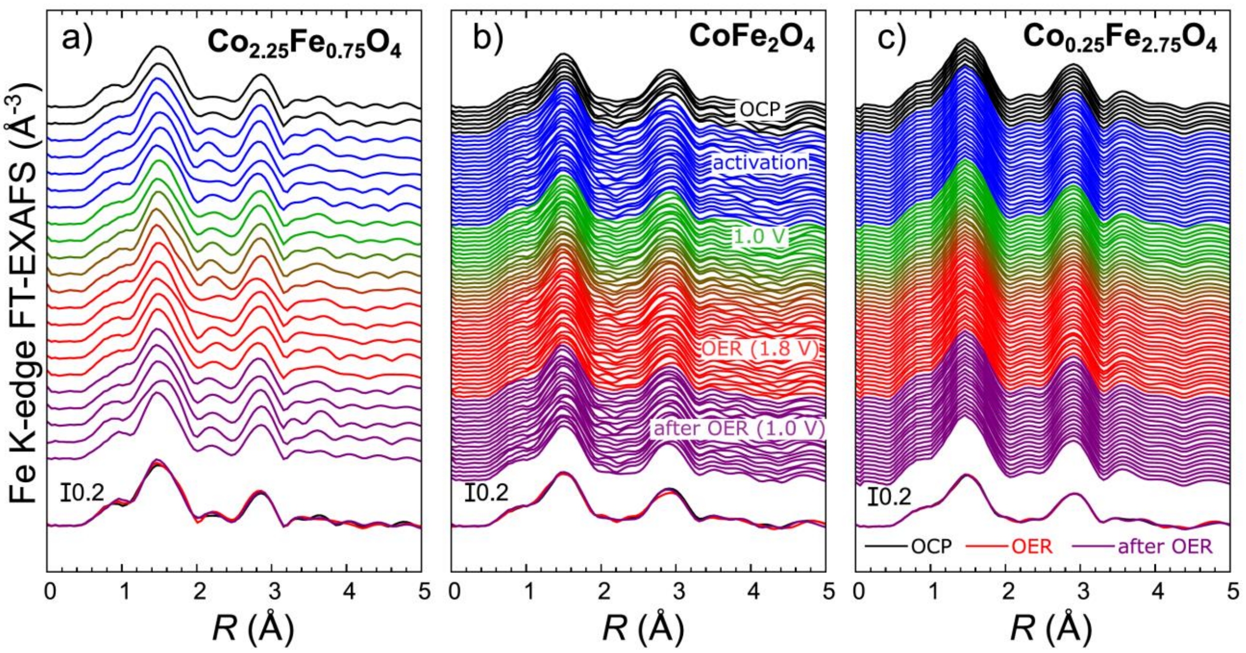

Figure S18. Evolution of Fourier-transformed Fe K-edge EXAFS spectra during the activation and under OER conditions at 1.8V_{RHE} for $Co_{2.25}Fe_{0.75}O_4$ (a), $CoFe_2O_4$ (b) and $Co_{0.25}Fe_{2.75}O_4$ (c) catalysts. Spectra are shifted vertically for clarity. Insets compare the representative spectra for the as-prepared samples, samples under OER conditions and for samples after OER. Fourier transform was carried out in the *k*-range between 1 and 12 $Å^{-1}$. Each depicted spectrum is obtained after averaging the QXAFS data collected within 50 s (for panels (b, c)), and within 200 s for spectra in panel (a).

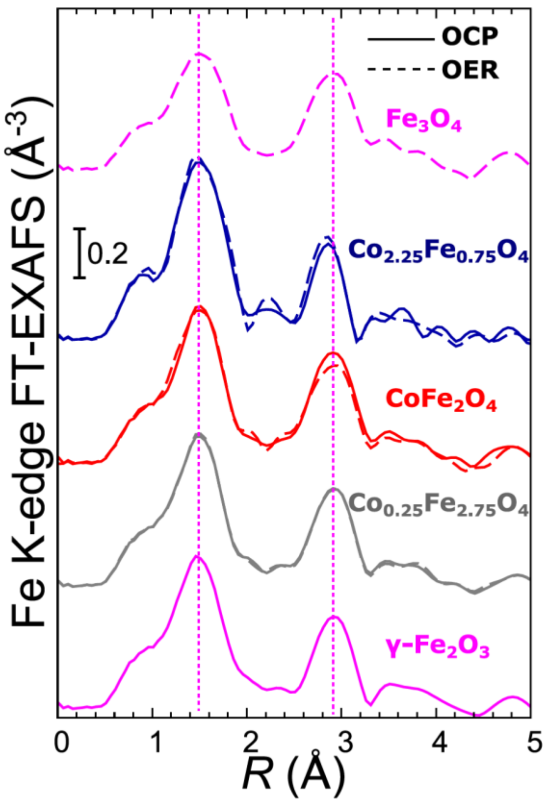

Figure S19. Comparison of Fourier-transformed Fe K-edge EXAFS spectra for all $Co_xFe_yO_z$ samples with different Co to Fe ratio. Spectra collected for the as-prepared samples under OCP, and for samples under OER conditions at $1.8V_{RHE}$ are shown, as well as the spectra for reference spinel-like oxides γ-Fe_2O_3 and Fe_3O_4. Fourier transform was carried out in the *k*-range between 1 and 12 $Å^{-1}$. Spectra are shifted vertically for clarity.

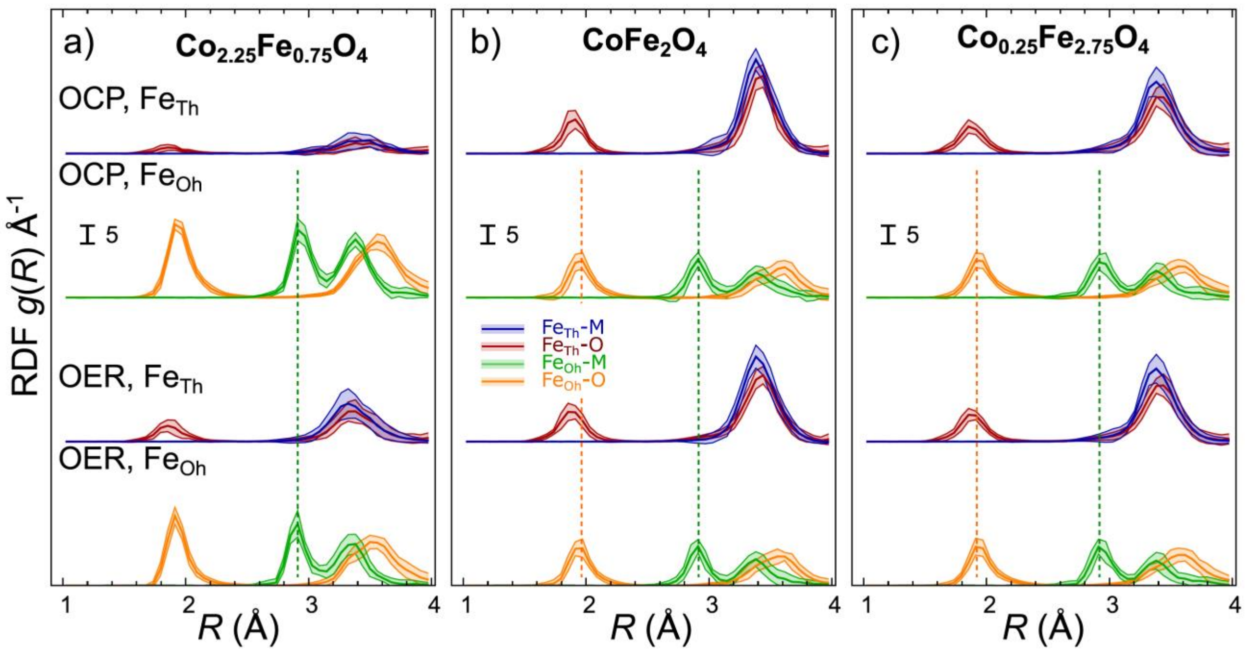

Figure S20. Partial RDFs extracted by NN from *operando* Fe K-edge EXAFS data for $Co_{2.25}Fe_{0.75}O_4$ (a), $CoFe_2O_4$ (b) and $Co_{0.25}Fe_{2.75}O_4$ (c). The plotted RDFs correspond to Fe-O and Fe-M contributions for tetrahedrally and octahedrally coordinated Fe sites for as-prepared samples under OCP conditions, and for samples under OER conditions. RDFs are shifted vertically for clarity.

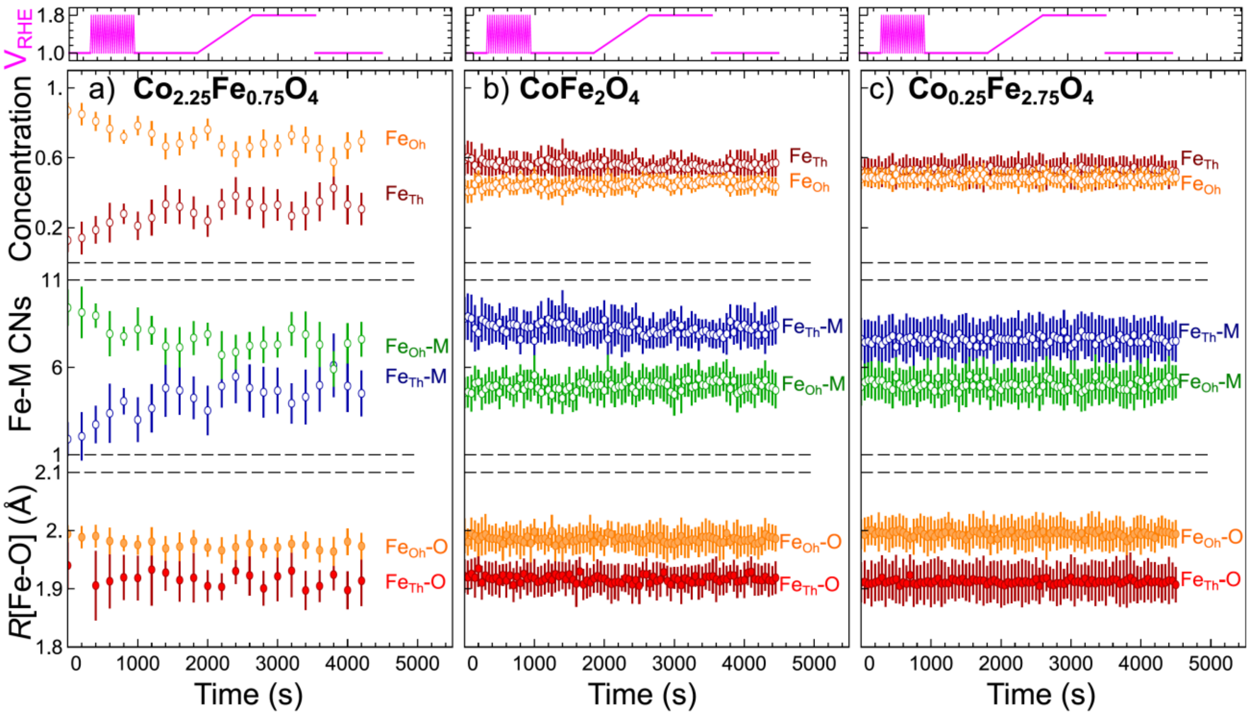

Figure S21. Evolution of Fe local structure parameters, extracted by integrating RDFs yielded by NN-EXAFS approach, during the activation and under OER conditions at 1.8V_{RHE} for $Co_{2.25}Fe_{0.75}O_4$ (a), $CoFe_2O_4$ (b) and $Co_{0.25}Fe_{2.75}O_4$ catalysts (c). The depicted quantities are (i) the concentrations of Fe_{Th} and Fe_{Oh} sites, obtained from the 1st shell apparent coordination numbers as $\widetilde{N}[Fe_{Th}-O]/4$ and $\widetilde{N}[Fe_{Oh}-O]/6$, respectively; (ii) the total Fe_{Th}-M and Fe_{Oh}-M apparent coordination numbers, obtained by integrating the Fe_{Th}-M and Fe_{Oh}-M partial RDFs in the *R*-range between 1 and 4 Å; and (iii) the average Fe_{Th}-O and Fe_{Oh}-O interatomic distances, obtained by integrating the Fe_{Th}-O and Fe_{Oh}-O partial RDFs in the *R*-range between 1 and 2.56 Å.

REFERENCES

(1) Martini, A.; Borfecchia, E. Spectral Decomposition of X-ray Absorption Spectroscopy Datasets: Methods and Applications. *Crystals* **2020**, *10* (8), 664.
(2) Martini, A.; Guda, A.; Guda, S.; Bugaev, A.; Safonova, O.; Soldatov, A. Machine learning powered by principal component descriptors as the key for sorted structural fit of XANES. *Physical Chemistry Chemical Physics* **2021**, *23* (33), 17873-17887.
(3) Timoshenko, J.; Frenkel, A. I. "Inverting" X-ray Absorption Spectra of Catalysts by Machine Learning in Search for Activity Descriptors. *ACS Catal.* **2019**, *9* (11), 10192-10211.
(4) Timoshenko, J.; Jeon, H. S.; Sinev, I.; Haase, F. T.; Herzog, A.; Cuenya, B. R. Linking the evolution of catalytic properties and structural changes in copper–zinc nanocatalysts using operando EXAFS and neural-networks. *Chemical Science* **2020**, *11* (14), 3727-3736.
(5) Hejral, U.; Timoshenko, J.; Kordus, D.; Luna, M. L.; Divins, N. J.; Widrinna, S.; Zegkinoglou, I.; Pielsticker, L.; Mistry, H.; Boscoboinik, J. A. Tracking the phase changes in micelle-based NiGa nanocatalysts for methanol synthesis under activation and working conditions. *Journal of Catalysis* **2022**, *405*, 183-198. Rüscher, M.; Herzog, A.; Timoshenko, J.; Jeon, H. S.; Frandsen, W.; Kühl, S.; Cuenya, B. R. Tracking heterogeneous structural motifs and the redox behaviour of copper–zinc nanocatalysts for the electrocatalytic CO 2 reduction using operando time resolved spectroscopy and machine learning. *Catalysis Science & Technology* **2022**, *12*, 3028-3043.
(6) Lewis, G.; Catlow, C. Potential models for ionic oxides. *Journal of Physics C: Solid State Physics* **1985**, *18* (6), 1149.
(7) Cooke, D. J.; Parker, S. C.; Osguthorpe, D. J. Calculating the vibrational thermodynamic properties of bulk oxides using lattice dynamics and molecular dynamics. *Physical Review B* **2003**, *67* (13), 134306.
(8) Erlebach, A.; Kurland, H.-D.; Grabow, J.; Müller, F. A.; Sierka, M. Structure evolution of nanoparticulate Fe 2 O 3. *Nanoscale* **2015**, *7* (7), 2960-2969.
(9) Hu, X. L.; Piccinin, S.; Laio, A.; Fabris, S. Atomistic structure of cobalt-phosphate nanoparticles for catalytic water oxidation. *ACS nano* **2012**, *6* (12), 10497-10504.
(10) Gale, J. D. GULP: A computer program for the symmetry-adapted simulation of solids. *Journal of the Chemical Society, Faraday Transactions* **1997**, *93* (4), 629-637. Gale, J. D.; Rohl, A. L. The general utility lattice program (GULP). *Molecular Simulation* **2003**, *29* (5), 291-341.
(11) Timoshenko, J.; Kuzmin, A.; Purans, J. EXAFS study of hydrogen intercalation into ReO_3 using the evolutionary algorithm. *J. Phys.: Condens. Matter* **2014**, *26* (5), 055401.

(12) Ankudinov, A. L.; Ravel, B.; Rehr, J. J.; Conradson, S. D. Real-space multiple-scattering calculation and interpretation of X-ray-absorption near-edge structure. *Phys. Rev. B* **1998**, *58* (12), 7565-7576.

(13) Timoshenko, J.; Kuzmin, A. Wavelet data analysis of EXAFS spectra. *Computer Physics Communications* **2009**, *180* (6), 920-925.
(14) *Wolfram Mathematica*; Wolfram Research, Inc.: Champaign, Illinois, 2022. https://www.wolfram.com/mathematica
(15) Timoshenko, J.; Duan, Z.; Henkelman, G.; Crooks, R.; Frenkel, A. Solving the structure and dynamics of metal nanoparticles by combining X-ray absorption fine structure spectroscopy and atomistic structure simulations. *Annual Review of Analytical Chemistry* **2019**, *12*, 501-522.

Gurman, S.; McGreevy, R. Reverse Monte Carlo simulation for the analysis of EXAFS data. *Journal of Physics: Condensed Matter* **1990**, *2* (48), 9463.
(16) Menard, L. D.; Wang, Q.; Kang, J. H.; Sealey, A. J.; Girolami, G. S.; Teng, X.; Frenkel, A. I.; Nuzzo, R. G. Structural characterization of bimetallic nanomaterials with overlapping x-ray absorption edges. *Physical Review B* **2009**, *80* (6), 064111.
